# Pain profiling of patients with temporomandibular joint arthralgia and osteoarthritis diagnosed with different imaging techniques

**DOI:** 10.1186/s10194-016-0653-6

**Published:** 2016-06-27

**Authors:** Simple Futarmal Kothari, Lene Baad-Hansen, Lars Bolvig Hansen, Niels Bang, Leif Hovgaard Sørensen, Helle Wulf Eskildsen, Peter Svensson

**Affiliations:** Section of Orofacial Pain and Jaw Function, Institute of Odontology and Oral Health, Aarhus University, Vennelyst Boulevard 9, DK-8000 Aarhus C, Denmark; Scandinavian Center for Orofacial Neurosciences (SCON), Aarhus University Hospital, Aarhus, Denmark; Department of Radiology, Aarhus University Hospital, Aarhus, Denmark; Department of Neuroradiology, Aarhus University Hospital, Aarhus, Denmark; Department of Dental Medicine, Karolinska Institutet, Huddinge, Sweden

**Keywords:** Temporomandibular joint arthralgia, Temporomandibular joint osteoarthritis, Quantitative sensory testing, Conditioned pain modulation, Cone-beam computed tomography, Magnetic resonance imaging, Ultrasonography, Somatosensory function

## Abstract

**Background:**

Clinical differentiation between pain mechanisms of temporomandibular joint (TMJ) arthralgia and osteoarthritis (OA) is challenging. The aims were to compare somatosensory function at the TMJs and conditioned pain modulation (CPM) effects between TMJ arthralgia and OA patients diagnosed clinically and based on different imaging techniques and age- and gender-matched healthy controls (*n* = 41).

**Methods:**

Patients (*n* = 58) underwent standard clinical examination and three different TMJ imaging modalities. After each examination, they were classified into arthralgia or OA based on the findings. TMJ region somatosensory testing was performed in all participants. Z-scores were calculated for patients based on healthy reference data. CPM was tested by comparing pressure pain thresholds (PPTs) at TMJ and thenar (control) before, during and after the application of painful and nonpainful cold stimuli. Data were analyzed using analyses of variance.

**Results:**

Somatosensory abnormalities were commonly detected in both patient groups. Assessment of somatosensory function at the TMJ revealed that arthralgia patients were less sensitive to warmth, cold and tactile stimuli than OA patients (*P* < 0.048). OA patients showed pressure hyperalgesia compared with arthralgia patients (*P* = 0.025). There was a significant CPM effect at both test sites during painful cold application in all groups (*P* < 0.001). There was no significant difference in the relative CPM effect between groups except for clinically diagnosed arthralgia patients showing reduced CPM effect compared with controls (*P* = 0.047).

**Conclusions:**

Pain profiles including somatosensory function differed between TMJ arthralgia and OA patients although CPM effects were similar in patients and controls. Thus, different TMJ pain conditions may share common pain mechanisms but the present study for the first time also indicated that differential pain mechanisms could be involved.

**Electronic supplementary material:**

The online version of this article (doi:10.1186/s10194-016-0653-6) contains supplementary material, which is available to authorized users.

## Background

Temporomandibular disorders (TMD) comprises a heterogenous group of complex disorders having varied and often multifactorial aetiologies [1, 2]. TMD are the most common chronic pain condition in the orofacial region [3], affecting the masticatory musculature, and the osseous and soft tissue components of the temporomandibular joint (TMJ). The Research Diagnostic Criteria for TMD (RDC/TMD) classifies TMD into three groups as I) myofascial pain, II) TMJ disc displacements and III) TMJ arthralgia, osteoarthritis (OA) and osteoarthrosis [4]. This study focused on the painful diagnoses of the third main category, i.e. TMJ pain. TMJ arthralgia according to the RDC/TMD is defined as spontaneous pain perceived from the TMJ region in addition to pain on palpation of the lateral pole or posterior attachment of the TMJ on the same side [4]. In contrast, TMJ OA involves pain in the TMJ associated with inflammation and degenerative processes of the bony components revealed by imaging techniques or coarse crepitation [4]. Although these criteria are internationally accepted, have been shown to be reliable, and recently have been updated to the Diagnostic Criteria for TMD (DC/TMD) [5–7], there are still major concerns about how to differentiate between the involved pain mechanisms in TMJ arthralgia and OA. A good understanding of underlying pain mechanisms may have significant implications for the rational management of pain and dysfunction.

Moreover, due to the diverse nature of TMD symptoms, patient evaluation with clinical examination alone is insufficient to fully assess the osseous and soft tissue components of the TMJ and often requires imaging to strengthen the diagnostic process [8, 9]. Also, TMJ OA is characterized by a gradual progressive destruction of articular tissues. With advanced degeneration, the subchondral cortical layer is lost and erosion and other radiographic signs of OA appear [10–12] enforcing the need of imaging for a valid diagnosis of OA. A variety of imaging modalities have been used to evaluate the TMJ. Cone beam computed tomography (CBCT) is used to assess only the osseous components of the joints [13], whereas magnetic resonance imaging (MRI) and ultrasonography (US) both allow visualization of the soft-tissue components and assessment of joint effusion and inflammation along with the assessment of osseous components [9].

Chronic TMJ pain has been associated with somatosensory disturbances such as increased pain sensitivity [14, 15]. Somatosensory sensitivity can be evaluated with quantitative sensory testing (QST) [16–18]. The German Research Network on Neuropathic Pain (DFNS) has introduced a standardized protocol of QST, where somatosensory profiles and LossGain scores based on Z-scores can be computed [17, 19]. The main hypothesis of this protocol is that detected patterns of sensory loss and gain of function can indirectly refer to underlying neurobiological mechanisms of altered pain sensitivity [20]. Several studies have described somatosensory function in myofascial TMD patients with patients showing increased sensitivity to thermal, mechanical and electrical stimuli [20–22] and a relatively few studies have also assessed somatosensory function in arthrogenous TMD patients [23, 24]. However, these studies did not employ the standardized German QST protocol. Moreover, recently, using the standardized DFNS protocol, we have shown that the majority of TMD pain patients exhibit somatosensory abnormalities [15]. However, so far no studies have evaluated somatosensory function in TMJ arthralgia and OA patients separately.

Endogenous pain modulation, involving supraspinal diffuse noxious inhibitory controls (DNIC), has been shown to be impaired in patients with idiopathic pain disorders such as fibromyalgia, tension type headache, painful osteoarthritis and TMD [25–27]. The function of the endogenous pain inhibitory systems in humans can be assessed by conditioned pain modulation (CPM) paradigms [28]. Studies evaluating the CPM effect in TMD patients show conflicting results; studies by King et al. and Oono et al. reported increased sensitivity to heat pain and mechanical pain, respectively, but failed to demonstrate CPM in TMD patients [27, 29], whereas, a significant CPM effect was demonstrated by application of a pain temporal summation paradigm by Garrett et al. and pressure pain by Kothari et al. [15, 30]. These studies included either myofascial or arthrogenous TMD patients or both but none of the studies compared CPM effects between different sub-categories of TMD.

Therefore, the aims of this study were (1) to compare the somatosensory function at and around the TMJ in TMJ arthralgia and OA patients diagnosed clinically without and in combination with different imaging techniques and (2) to examine whether the CPM differs between the TMJ arthralgia and OA patients diagnosed clinically without and in combination with different imaging modalities and also in comparison with healthy controls.

## Methods

### Participants

Fifty-eight TMD pain patients (48 women and 10 men; mean ± SEM age: 37.2 ± 1.9 years, age range 20–74 years) and 41 age- and gender-matched healthy participants (30 women and 11 men; mean ± SEM age: 32.0 ± 1.9 years, age range 20–61 years) were included. The patients were recruited from the Section of Orofacial Pain and Jaw Function, Department of Dentistry, Aarhus University. The healthy participants were invited to participate in the study by posting advertisement on web pages and flyers at and around the university regarding the study. The study was conducted at the Institute of Odontology and Oral Health and the imaging techniques such as MRI and high-resolution ultrasonography (HR-US) were performed at Aarhus University Hospital. Prior to the study, all the participants were clinically examined according to the RDC/TMD axis I protocol [4]. The healthy participants included had no signs or symptoms of TMD or musculoskeletal or rheumatologic disease or any previous injuries interfering with the normal somatosensory function. The inclusion criteria for TMD pain patients were: (a) Adults over the age of 18; (b) TMD pain patients belonging to group IIIa (i.e., TMJ arthralgia-spontaneous pain or pain on movements in the TMJ, and pain on palpation of the lateral pole or posterior attachment of the TMJ on the same side) or group IIIb (i.e., TMJ osteoarthritis (OA)-TMJ arthralgia plus either coarse crepitus in the joint or degenerative changes in the joint supported by CBCT findings) from RDC/TMD [4] (c) Patients reporting TMJ pain longer than 3 months. All the included TMD pain patients were then divided into 2 groups: a) TMJ arthralgia and b) TMJ OA based on the clinical examination. Furthermore, patients with additional diagnoses of myofascial pain (group I) and disk displacements (group II) to group IIIa or IIIb from RDC/TMD protocol were also accepted [4]. Exclusion criteria included TMJ pain conditions related to acute trauma, rheumatoid arthritis or other generalized joint conditions and contraindications to MRI such as claustrophobia, metal prostheses, pregnancy, and pacemakers. The study mainly focused on patients with TMJ pain (i.e. TMJ arthralgia and OA), however, as mentioned above, patients with co-morbid diagnoses of group I and II from RDC/TMD were also included. Therefore the term “TMD pain patients” has been used throughout the manuscript wherever needed. Out of 58 TMD pain patients, 35 patients had pain unilaterally and 23 patients had pain bilaterally at the TMJ. All participants gave their written informed consent prior to study participation, in accordance with the Helsinki Declaration. The study was approved by the local ethics committee in Central Denmark Region, Denmark.

### Self-reported measures of pain

Characteristic pain intensity (CPI) is a self-report measure derived from the RDC/TMD history questionnaire [4]. It reflects current pain, average pain, and worst pain in the jaw during the last 6 months. The resulting CPI score ranges from 0 to 100, with 100 being the most painful. In addition, the Graded Chronic Pain Scale (GCPS) consisting of measures for pain intensity and pain-related disability was also applied [4]. This scale is divided into following scores: 0, no disability; I, low disability, low intensity; II, low disability, high intensity; III, high disability, moderately limiting; and IV, high disability, severely limiting [4]. For a detailed description, refer to the original RDC/TMD publication [4].

### TMJ imaging

All the TMD pain patients underwent CBCT, MRI and HR-US of both TMJs in order to evaluate the presence of degenerative changes indicating OA. After the TMJ imaging, they were divided into two groups: TMJ arthralgia or OA. Soft-tissue changes/pathology such as synovial hypertrophy and presence of vascularization which were considered signs of OA, disc deformity, disc displacements and presence or absence of effusion were also investigated using MRI and HR-US. TMJ OA was diagnosed on MRI and CBCT based on the image analysis criteria developed by Ahmed et al. [31]. These criteria are reliable for diagnosing osseous and nonosseous components of TMJ using CT and MRI respectively. According to the criteria, deformation due to subcortical cyst, surface erosion, osteophyte, or generalized sclerosis are considered signs of TMJ OA [31]. However, no reliable and standardized imaging criteria were found for diagnosing TMJ OA on US. Therefore, in addition to the image analysis criteria proposed by Ahmed et al. for TMJ OA [31], synovial hypertrophy and presence of vascularization in the synovium, both indicating synovitis were also considered as signs of TMJ OA on US. This was based on the criteria used for diagnosing OA of other joints of the body like the knee [32]. HR-US was also performed on ten healthy participants in order to measure the normal thickness of the synovium. The radiologists were blinded to the clinical diagnosis of the patients as well as did not have any information about the results of the other imaging modalities.

### Cone beam computed tomography

The CBCT images of the bilateral TMJ were obtained with a Scanora 3D unit (Soredex Oy, Tuusula, Finland) with voltage set at 90 kV, current at 13 mA and an exposure time of 23 s. The voxel size was 0.20 mm. The effective dose was approximately 70 μSv and the field of view (FOV) was 7.5 × 14.5 cm. The patients were seated in the unit with a chin-rest to stabilize the mandible, and two vertical plastic bars (one on each side) to support the position of the head. The DICOM datasets containing the volumetric images were then imported into a reconstruction software-OnDemand 3D (Cybermed, Seoul, South Korea), and each one was adjusted to present optimal image characteristics; i.e. windowing (values for the center level (L) and band width (W) of the displayed shades of gray) of the image sets was adjusted (L = 722 and W = 3494) [33]. In this software, axial, coronal, and sagittal cross-sectional images (0.25 mm thick) were dynamically displayed on a 24-in. Dell LED screen, with a resolution of 1280 × 1024 pixels, in a dark room. One of the two independent experienced oral and maxillofacial radiologists, who routinely interpreted TMJ CBCT images, rated the images for degenerative changes in the TMJs.

### Magnetic resonance imaging

All MRI images were obtained on 1.5-T units (Magnetom Avanto; Siemens, Erlangen, Germany) using surface coils. On the sagittal plane, both the proton density-weighted image sequence (PDWI) and T_2_ weighted image sequence (T_2_WI) were obtained: repetition time (TR), 2800 ms; echo time (TE1), 14 ms; TE2, 82 ms; field of view (FOV), 140 × 140 mm; slice thickness, 3 mm; interslice gap, 0.3 mm; number of images, 7. On the coronal plane, only T1 weighted turbo spin echo images were obtained in the closed mouth position: TR, 717 ms; TE, 12 ms; field of view (FOV), 140 × 140 mm; slice thickness, 3 mm; interslice gap, 0.3 mm; number of images, 7. The coronal images were obtained in a plane parallel to the long axis of the mandibular condyles [34]. Sagittal images were obtained in a plane perpendicular to the long axis of the mandibular condyles [34]. The sagittal images were obtained in the closed mouth and maximal mouth opening positions. The MRI images were interpreted by one of the two experienced neuroradiologists having more than 20 years of experience. Findings compatible with degenerative changes indicative of OA were recorded.

### High-resolution ultrasonography

Ultrasound of TMJ was performed with a high-resolution (18–5 MHz) linear array transducer (Hitachi Ascendus Hi Vision, Japan). Patients were placed in a supine position. The transducer was positioned over the TMJ, parallel and inferior to the zygomatic arch for an axial view and parallel to the mandibular ramus for a coronal view [35]. The transducer was tilted out until an optimal visualization of the joint was obtained. Static scans were obtained with the closed-mouth position and maximum-mouth opening position, and dynamic scans were obtained during mouth opening. The width of the synovium was measured as the distance between the echogenic joint capsule and the articular surface. It was measured in both open and closed mouth positions. Presence of vascularization in the synovium was graded from 0 to 4 (Grade 0 = no vascularization; Grade 1 = 1 blood vessel; Grade 2 = < 25 % of synovium filled with blood vessels; Grade 3 = 25–50 % of synovium filled with blood vessels; Grade 4 = > 50 % of synovium filled with blood vessels) [36]. Each scanning was performed and interpreted by one of two radiologists having more than 20 years of experience in US. All images were evaluated for degenerative changes of the TMJ compatible with OA.

### Quantitative sensory testing

A standardized battery of QST was performed according to the German protocol [17]. The QST battery consists of seven tests measuring 13 parameters that cover relevant nerve function [20]. For a detailed description of the protocol, see Rolke et al. [17]. In summary, the protocol investigates the following sensory functions: *Thermal thresholds*: cold detection (CDT), warmth detection (WDT), cold pain (CPT), heat pain (HPT), and thermal sensory limen (TSL); *Mechanical thresholds*: mechanical detection (MDT), vibration detection (VDT), mechanical pain (MPT), and pressure pain (PPT); *Stimulus-response functions*: mechanical pain sensitivity (MPS), dynamic mechanical allodynia (DMA), wind-up ratio: pain summation to repetitive pinprick (WUR), and paradoxical heat sensations (PHS) during the thermal limen procedure [17].

QST was performed on the skin overlying the TMJ on both sides in all patients and healthy controls. In patients, the most painful side was defined as the test site and the non-painful or less painful side was defined as the control site. In healthy controls, the dominant side was defined as the test site. The detailed methodology is included in the Additional file 1.

### Conditioned pain modulation (CPM)

The protocol for CPM used in this study was similar to the one used in our previous study [15]. Using a Somedic pressure algometer, PPT was measured thrice at three separate time points i.e., before (baseline), during and after the application of a noxious conditioning cold stimulus (cold pressor test involving immersion of the subject’s foot in ice water maintained at 2–4 °C) [37] and non-noxious conditioning stimulus (involving neutral water maintained at 26–28 °C) [15, 38]. PPT ratings were first obtained at baseline on the test site of TMJ (i.e., most painful side of TMJ) and the thenar muscle of the dominant hand (control). Immediately after measuring PPT at baseline, participants underwent the cold pressor test. During this test, participants were asked to immerse their dominant leg up to the ankle, in a cold water bath maintained at 2–4 °C for 3 min. PPT was again measured at the same sites (TMJ and thenar muscle) after 1.5 min of leg immersion. During the leg immersion, the participants were also asked to rate the pain intensity and the unpleasantness on two separate NRSs ranging from 0 (“no pain” / “not at all unpleasant”) to 100 (“the most intense pain imaginable” / “the most unpleasantness imaginable”) [15, 39]. After 3 mins, the participants were asked to remove their leg from the water. The PPT was re-assessed at TMJ and thenar muscle soon after the cold pain disappeared [15]. The order of assessment of PPTs at TMJ and thenar muscle were randomized throughout the procedure [15].

**Fig. 1:**
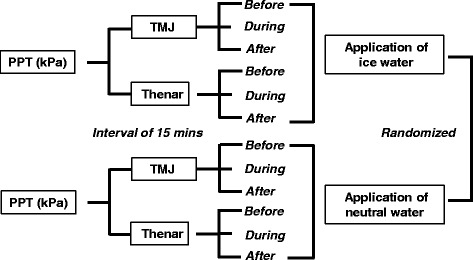
Overview of the study design for conditioned pain modulation (CPM). PPT, pressure pain threshold; TMJ, temporomandibular joint

Similar methodology was repeated using neutral water/non-painful water as conditioning stimulus after an interval of 12–15 min [15]. Here, again PPT measurements were taken thrice at the TMJ and thenar before, during and after the application of the conditioning stimulus. Participants also rated the pain intensity and unpleasantness on the 0–100 NRS scales during the leg immersion in neutral water. The sequence of immersion of leg in ice or neutral water was randomized [15] (Fig. 1).

### Data evaluation and statistical analysis

#### Z-transformation of QST data

All QST parameters except the CPT, HPT, VDT and PHS were normally distributed in log-space and were transformed logarithmically before statistical analysis [17]. A small constant (0.1) was added to all pain ratings (MPS, DMA) prior to calculating the logarithm in order to avoid a loss of zero values [15]. To compare a single patient’s QST data profile with the group mean of age- and gender-matched healthy controls (mean data from left and right TMJ pooled), the data from the individual patients were Z-transformed for each single parameter by using the following expression: Z-score = (Value_single patient_ – Mean_controls_) / SD_controls_ [40]. After Z-transformation, all patients’ QST data were presented as standard normal distributions (zero mean, unit variance) [20]. Values were adjusted for signs in such a way that positive Z-scores indicated gain of somatosensory function when the patient was more sensitive to the tested stimuli compared with controls (hyperesthesia, hyperalgesia, allodynia), and negative Z-scores indicated loss of function, referring to a lower sensitivity of the patient (hypoesthesia, hypoalgesia) [19]. A Z-score of zero represented an individual value corresponding to the group mean of the healthy controls [41]. The Z-scores of 0 ± 1.96 represents the range that would be expected to include 95 % of the healthy control subject data. Therefore, any Z-scores outside the 95 % confidence interval (CI) of the healthy control data (i.e. Z-score < −1.96 or >1.96) were considered as absolute abnormalities [19]. In addition, the side-to-side differences of each QST parameter were compared with the 95 % CI of the side-to-side differences of the healthy reference group [19]. If the side-to-side differences were larger than the upper limit of the 95 % CI of the healthy reference group, the value was considered as a relative abnormality [17, 19, 42]. In accordance with Maier et al. [19] both absolute and relative abnormalities were taken into account in order to assess frequencies of loss and gain of somatosensory function.

#### Assessment of somatosensory loss and gain of function

The LossGain coding system was applied in order to display combinations of sensory abnormalities in the patients [19, 42]. Here both absolute and relative abnormalities are combined into one single sensitivity score per patient. This coding system combines a score of somatosensory loss of function (L0, L1, L2, or L3) with a score of somatosensory gain of function (G0, G1, G2, or G3) [19, 42, 43]. The number ‘1’ in L1 and G1 indicates somatosensory abnormality related to the thermal modalities alone, ‘2’ in L2 and G2 indicates abnormality related to mechanical modalities alone and ‘3’ indicates abnormalities related to both thermal and mechanical modalities (mixed) [19, 43]. If measures of thermal and/ or mechanical detection (CDT, WDT, MDT or VDT) were abnormal on the affected side in comparison with the healthy reference data (absolute abnormality) or if abnormally large side-to-side differences were detected (relative abnormality), it was recorded as one of the following: L1 - isolated loss of small fiber function (if abnormal thermal detection thresholds (CDT or WDT) alone); L2 - isolated loss of large fiber function (if abnormal mechanical detection thresholds (MDT or VDT) alone); or L3 - mixed loss of function (if loss of both small and large fiber function) [19, 42]. Similarly, for somatosensory gain, thermal hyperalgesia (G1) was recorded, if somatosensory gain of function in cold or heat pain thresholds (CPT or HPT) were found (absolute or relative abnormality). Mechanical hyperalgesia (G2) was recorded, if gain of function (absolute or relative abnormality) was detected for mechanical pain threshold (MPT), mechanical pain sensitivity (MPS), pressure pain threshold (PPT) or if the dynamic mechanical allodynia (DMA) score exceeded 0 [19, 42, 43]. Mixed gain (G3) was recorded in patients with gain of both thermal and mechanical somatosensory function. L0 and G0 were scored if there was no detection of loss or gain of somatosensory function respectively [19].

#### Assessment of synovial thickness

The thickness of the synovial membrane of the TMJ was measured in all the patients and ten included healthy controls by HR-US. It was measured in both opened and closed mouth positions at both TMJs. The values obtained were Z-transformed to compare each patient’s synovial thickness with the group mean of the healthy controls. For this, the expression: Z-score = (Value_single patient_ – Mean_controls_) / SD_controls_ was applied to the individual patient’s synovial thickness measurement at each side during both the positions. Here, the Z-transformation performed was identical to the one performed for QST parameters, which is mentioned above [17]. However, upper-tailed Z-scores (i.e., one-tailed with positive values) were calculated in contrast to the two-tailed Z-scores that were used for evaluating QST parameters. Upper-tailed Z-scores were used as the thickness of synovial membrane can only be considered abnormal if it is significantly thicker than the reference group [44]. A Z-score of zero indicated an individual value corresponding to the group mean of the healthy controls. The Z-scores of below 1.645 represented the range that would be expected to include 95 % of the healthy control reference data [45]. Therefore, any Z-score outside the 95 % CI of the healthy control data (i.e. Z-score > 1.645) was considered as abnormal synovial thickness or synovial hypertrophy. Synovial thickness was measured in millimeters. Further, using the clinical diagnosis plus CBCT as the reference standard, sensitivity and specificity of the HR-US for osteoarthritic changes were calculated.

#### Statistics

Age and gender distribution between subject groups (TMD pain patients and healthy controls) were analyzed by unpaired *t*-test and *χ*^2^-test respectively.

QST data for each parameter were compared between the subject groups (patients/controls) and sides using a two-way analysis of variance (ANOVA) with sides (test site/control site) as within-subjects factor and group (arthralgia patients/OA patients/controls) as between-subjects factor. This analysis was carried out each time after the patients were diagnosed as belonging to either the arthralgia or OA group based on: 1. Clinical examination alone, clinical examination in combination with each of the following imaging modalities: 2. CBCT, 3. MRI and 4. HR-US. Further, to compare the QST data for each parameter between the “pure” arthralgia patients (i.e., patients with no crepitus or abnormalities/degenerative changes on any imaging modality) and OA patients, showing degenerative changes on all 3 imaging modalities (i.e., combined diagnosis), two-way ANOVA with group (arthralgia patients/OA patients/controls) as between-subjects factor and sides (test site/control site) as within-subjects factor was employed. To evaluate the difference between the painful and non-painful control site in patients regardless of diagnosis, QST data of each parameter were compared using paired t-tests. Further, to evaluate if comorbid diagnoses of myofascial pain across arthralgia and OA patient groups would have had influence on the somatosensory function, χ^2^-test was used to analyze the distribution of group I diagnoses across the two patient groups diagnosed clinically at the test site. Post-hoc comparisons were performed using Tukey HSD tests with correction for multiple comparisons.

As in the QST data analysis, the CPM data was also repeatedly analysed between the groups (arthralgia patients/OA patients/controls), based on the diagnosis of patients after clinical examination without and in combination with each imaging technique. For each analysis, the CPM effect was tested by performing a four-way ANOVA on absolute PPT values. The factors in the ANOVA were: subject group (arthralgia patients/OA patients/controls) as between group factor, site (TMJ, thenar), session (neutral water, water maintained at 2–4 °C), and time (before, during, after) as repeated measures. When appropriate, the ANOVAs were followed by post-hoc Tukey HSD tests with adjustment for multiple comparisons. Relative changes in PPT during the application of the noxious conditioning cold stimulus were calculated in order to control for baseline differences between the subject groups at both the sites (relative PPT changes = (PPT during application of noxious cold stimulus-PPT at baseline)/(PPT at baseline) ×100). Then, the relative changes in PPT were analyzed using unpaired t-tests to assess the effect of CPM between subject group at TMJ and thenar. Further, the CPM effect between pure arthralgia, OA patients showing degenerative changes on all 3 imaging modalities and healthy controls was also analysed in the same way. Paired t-tests were used to compare the NRS scores for pain intensity and unpleasantness during the application of the conditioning stimuli for both the groups (TMD pain patients and healthy controls).

All data are presented as mean values and standard errors of mean (SEM). For all tests, values of *P* < 0.05 were considered as statistically significant. Data were analyzed using Statistica software for windows (StatSoft Inc., USA).

## Results

### Patient description

There was no significant difference in the age and gender distribution between the TMD pain patients and the healthy controls (age: *P* = 0.061; gender: *P* = 0.294).

**Fig. 2:**
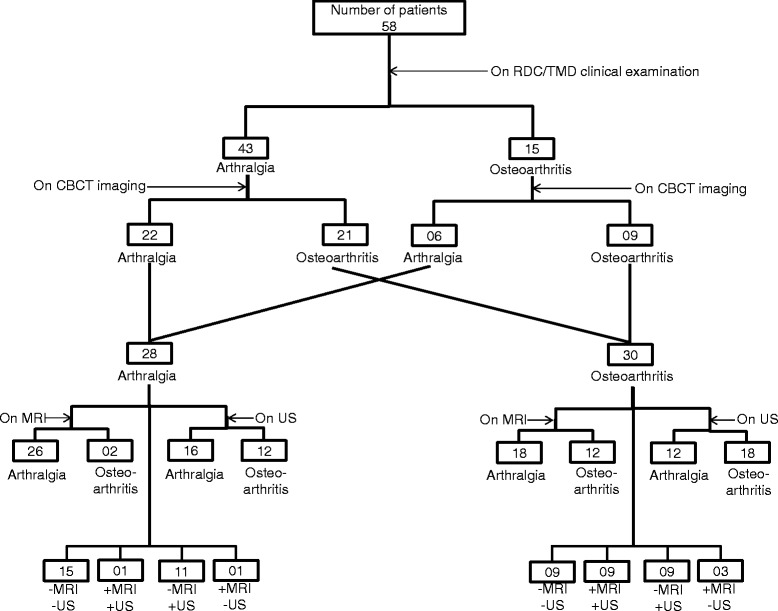
A flow chart depicting how the included TMD pain patients were classified into TMJ arthralgia and TMJ osteoarthritis (OA) patients based on findings from different examination methods at most painful TMJ. All the patients underwent RDC/TMD clinical examination and three different imaging modalities. Initially, patients were grouped into arthralgia and OA patients based on the findings from RDC/TMD clinical examination. After this, patients showing degenerative changes on CBCT imaging along with the presence of coarse crepitus at the joint during the clinical examination were classified as OA patients and those without the degenerative changes irrespective of coarse crepitus at the joint were classified as arthralgia patients. These arthralgia and OA patients were further classified based on the presence or absence of degenerative changes on MRI and HR-US as OA and arthralgia patients respectively. Furthermore, agreement between MRI and HR-US findings for the diagnosis of arthralgia and OA is also presented, where “−” indicated no degenerative changes seen and “+” indicated degenerative changes found. Accordingly,−MRI−US indicates patients belonging to the arthralgia group and + MRI + US indicates patients belonging to OA group. RDC/TMD, Research Diagnostic Criteria for Temporomandibular disorders; TMJ, temporomandibular joint; CBCT, cone beam computerized tomography; MRI, magnetic resonance imaging; US, high resolution ultrasonography, HR-US, high resolution ultrasonography

All the patients, indeed, had TMD pain according to the clinical RDC/TMD protocol. At the most painful TMJ, based on the clinical examination alone, there were 43 TMJ arthralgia patients (group IIIa) and 15 TMJ OA patients (group IIIb). CBCT imaging revealed that out of 43 patients with a clinical arthralgia diagnosis, 21 had degenerative changes in the TMJ. Thus, these patients were re-classified as OA of the TMJ (group IIIb) based on imaging findings. Also, out of 15 OA patients diagnosed clinically, only 9 showed degenerative changes on CBCT. Thus, these patients were classified again as OA patients. The other 6 patients without degenerative changes on CBCT, regardless of the coarse TMJ crepitus were reclassified as arthralgia patients. Therefore, all the included TMD pain patients were classified as 28 TMJ arthralgia and 30 TMJ OA based on clinical examination plus CBCT findings. These patients were further classified into OA and arthralgia based on the presence or absence of degenerative changes respectively on MRI and HR-US. Concordance between the MRI and HR-US for the diagnosis of OA and arthralgia was also noted. A flow chart showing the diagnosis of patients as belonging to the arthralgia or OA group based on clinical examination and different imaging techniques is presented in Fig. 2. Also, a detailed description of the clinical characteristics of the TMD pain patients according to RDC/TMD axis I diagnoses is presented in Additional file 2: Table S1. Comorbid myofascial pain diagnoses seen in group IIIa and IIIb patients diagnosed clinically at the most painful site were 35 and 12 respectively (χ^2^ test: *P* = 0.905).

As mentioned earlier, out of 58 patients (116 joints), 23 patients (46 joints) had pain bilaterally and 35 patients (35 joints) had pain unilaterally at the TMJ. Therefore, to get a better picture of the number of joints involved, after clinical diagnosis, they were divided into 3 groups-healthy joints (35 joints), joints with arthralgia (63 joints) and joints diagnosed as having OA (18 joints). A detailed description of the classification of joints is given in Additional file 1 and presented in Additional file 3: Figure S1.

### Self-reported pain

The mean CPI (0–100) of the TMD pain patients was 61.1 ± 2.2, whereas none of the healthy controls had pain in the TMJ area. The number of patients classified based on GCPS were: grade I-12 (20.7 %), grade II-34 (58.6 %), grade III-11 (19.0 %) and grade IV-1 (1.7 %).

### Hard and soft tissue findings on imaging

Degenerative changes of hard tissue origin such as erosion, osteophytes, generalized sclerosis and subcortical cyst were seen in 51.6, 19.0, and 12.0 % of patients at the test site and 43.0, 8.6, and 8.6 % of patients at the control site on CBCT, MRI and HR-US respectively. On HR-US, soft tissue changes such as synovial hypertrophy at closed mouth position and open mouth position were seen in 19.0 and 32.7 % at the test site, respectively, and 15.5 % in both the positions at the control site of patients. On MRI, synovial hypertrophy was seen in 8.6 % of patients at the test site and 0 % at the control site. Effusion was seen in 6.9 % of patients at the test site and 1.7 % of patients at the control site on both HR-US and MRI. Further, vascularization was seen in 15.5 % at the test site and 10.3 % at the control site of patients on HR-US. In addition, on MRI, disc displacements were seen in 20.6 and 17.2 %, narrow joint space in 13.8 and 5.2 % and narrowed disc in 27.5 and 13.8 % of patients at the test site and control site respectively. The sensitivity and specificity of HR-US for the diagnosis of OA is given in Additional file 1.

### Somatosensory function

The frequencies of absolute abnormalities of Z-scores for each QST parameter for controls, arthralgia and OA patients classified based on clinical examination, presence or absence of degenerative changes on CBCT, MRI and HR-US and combined examinations (i.e., pure arthralgia and OA diagnosed as OA on all imaging techniques) are shown in Table 1. The most frequent somatosensory absolute abnormalities (outside 95 % CI of reference data) at the test site found in both arthralgia and OA patients, diagnosed regardless of the examination modalities used were (in order of frequency): somatosensory gain with regard to PPT and MPT and somatosensory loss with regard to MDT and WDT. However, with regards to gain of function, in arthralgia patients both HPT and WUR occurred as the third most frequent somatosensory abnormality and in OA patients HPT occurred as the third and WUR occurred as the fourth most frequent somatosensory abnormality.

**Table 1:** Frequency (%) of TMJ arthralgia and osteoarthritis patients and healthy reference controls showing Z-score values outside the reference 95 % confidence interval of the reference data (−1.96 < Z < 1.96) at most painful TMJ

		^a^Clinical Diagnosis		^a^After CBCT		^a^After MRI		^a^After HR-US		^a^Combined	
	Controls (*n* = 41)	Art (*n* = 43)	OA (*n* = 15)	Art (*n* = 28)	OA (*n* = 30)	Art (*n* = 44)	OA (*n* = 14)	Art (*n* = 28)	OA (*n* = 30)	Art (*n* = 15)	OA (*n* = 9)
QST parameter	*n* (%)	*n* (%)	*n* (%)	*n* (%)	*n* (%)	*n* (%)	*n* (%)	*n* (%)	*n* (%)	*n* (%)	*n* (%)
CDT											
>1.96	0 (0.0)	0 (0.0)	0 (0.0)	0 (0.0)	0 (0.0)	0 (0.0)	0 (0.0)	0 (0.0)	0 (0.0)	0 (0.0)	0 (0.0)
<−1.96	2 (4.9)	2 (4.7)	2 (13.3)	2 (7.2)	2 (6.6)	3 (6.8)	1 (7.1)	4 (14.3)	0 (0.0)	2 (13.3)	0 (0.0)
WDT											
>1.96	0 (0.0)	0 (0.0)	0 (0.0)	0 (0.0)	0 (0.0)	0 (0.0)	0 (0.0)	0 (0.0)	0 (0.0)	0 (0.0)	0 (0.0)
<−1.96	2 (4.9)	9 (20.9)	2 (13.3)	3 (10.8)	8 (26.6)	7 (15.9)	4 (28.4)	8 (28.6)	3 (10.0)	2 (13.3)	1 (11.1)
TSL											
>1.96	0 (0.0)	1 (2.3)	0 (0.0)	0 (0.0)	1 (3.3)	0 (0.0)	1 (7.1)	0 (0.0)	1 (3.3)	0 (0.0)	1 (11.1)
<−1.96	1 (2.4)	3 (7.0)	2 (13.3)	3 (10.8)	2 (6.7)	4 (9.1)	1 (7.1)	5 (18.0)	0 (0.0)	3 (20.0)	0 (0.0)
CPT											
>1.96	0 (0.0)	2 (4.6)	2 (13.3)	2 (7.2)	2 (6.7)	3 (6.8)	1 (7.1)	2 (7.2)	2 (6.6)	1 (6.7)	1 (11.1)
<−1.96	0 (0.0)	0 (0.0)	0 (0.0)	0 (0.0)	0 (0.0)	0 (0.0)	0 (0.0)	0 (0.0)	0 (0.0)	0 (0.0)	0 (0.0)
HPT											
>1.96	0 (0.0)	5 (11.6)	3 (20.0)	2 (7.2)	6 (20.0)	4 (9.1)	4 (28.4)	3 (10.8)	5 (16.7)	1 (6.7)	4 (44.4)
<−1.96	0 (0.0)	2 (4.6)	2 (13.3)	2 (7.2)	2 (6.7)	3 (6.8)	1 (7.1)	3 (10.8)	1 (3.3)	1 (6.7)	0 (0.0)
MDT											
>1.96	0 (0.0)	0 (0.0)	0 (0.0)	0 (0.0)	0 (0.0)	0 (0.0)	0 (0.0)	0 (0.0)	0 (0.0)	0 (0.0)	0 (0.0)
<−1.96	1 (2.4)	10 (23.3)	5 (33.3)	7 (25.0)	8 (26.6)	12 (27.3)	3 (21.4)	10 (35.7)	5 (16.7)	5 (33.3)	2 (22.2)
MPT											
>1.96	2 (4.9)	10 (23.2)	4 (26.6)	5 (18)	9 (30.0)	9 (20.5)	5 (35.5)	5 (18.0)	9 (30.0)	4 (26.6)	5 (55.5)
<−1.96	0 (0.0)	0 (0.0)	0 (0.0)	0 (0.0)	0 (0.0)	0 (0.0)	0 (0.0)	0 (0.0)	0 (0.0)	0 (0.0)	0 (0.0)
MPS											
>1.96	3 (7.2)	3 (7.0)	1 (6.7)	2 (7.2)	2 (6.7)	3 (6.8)	1 (7.1)	3 (10.8)	1 (3.3)	1 (6.7)	0 (0.0)
<−1.96	0 (0.0)	0 (0.0)	0 (0.0)	0 (0.0)	0 (0.0)	0 (0.0)	0 (0.0)	0 (0.0)	0 (0.0)	0 (0.0)	0 (0.0)
WUR											
>1.96	1 (2.4)	5 (11.6)	2 (13.3)	4 (14.4)	3 (10.0)	4 (9.1)	3 (21.3)	3 (10.8)	4 (13.3)	1 (6.7)	1 (11.1)
<−1.96	0 (0.0)	0 (0.0)	0 (0.0)	0 (0.0)	0 (0.0)	0 (0.0)	0 (0.0)	0 (0.0)	0 (0.0)	0 (0.0)	0 (0.0)
VDT											
>1.96	0 (0.0)	0 (0.0)	0 (0.0)	0 (0.0)	0 (0.0)	0 (0.0)	0 (0.0)	0 (0.0)	0 (0.0)	0 (0.0)	0 (0.0)
<−1.96	1 (2.4)	2 (4.6)	1 (6.7)	1 (3.6)	2 (6.7)	2 (4.5)	1 (7.1)	2 (7.2)	1 (3.3)	1 (6.7)	0 (0.0)
PPT											
>1.96	0 (0.0)	24 (55.8)	10 (66.6)	17 (60.7)	17 (56.7)	25 (56.8)	9 (64.3)	14 (50.0)	20 (66.7)	9 (60.0)	6 (66.7)
<−1.96	0 (0.0)	0 (0.0)	0 (0.0)	0 (0.0)	0 (0.0)	0 (0.0)	0 (0.0)	0 (0.0)	0 (0.0)	0 (0.0)	0 (0.0)

*Abbreviations*: *TMJ* temporomandibular joint, *QST* quantitative sensory testing, *Art* arthralgia patients, *OA* osteoarthritis patients, *CBCT* Cone beam computed tomography, *MRI* Magnetic resonance imaging, *HR-US* high resolution ultrasonography, *CDT* cold detection threshold, *WDT* warm detection threshold, *TSL* thermal sensory limen, *CPT* cold pain threshold, *HPT* heat pain threshold, *MDT* mechanical detection threshold, *MPT* mechanical pain threshold, *MPS* mechanical pain sensitivity, *WUR* windup ratio, *VDT* vibration detection threshold, *PPT* pressure pain threshold

^a^Patients were classified into TMJ arthralgia and osteoarthritis based on the diagnosis made after each examination modality (i.e., clinical examination, CBCT, MRI and HR-US and combined examinations (i.e., based on the presence or absence of degenerative changes on all imaging modalities at TMJ)

**Fig. 3:**
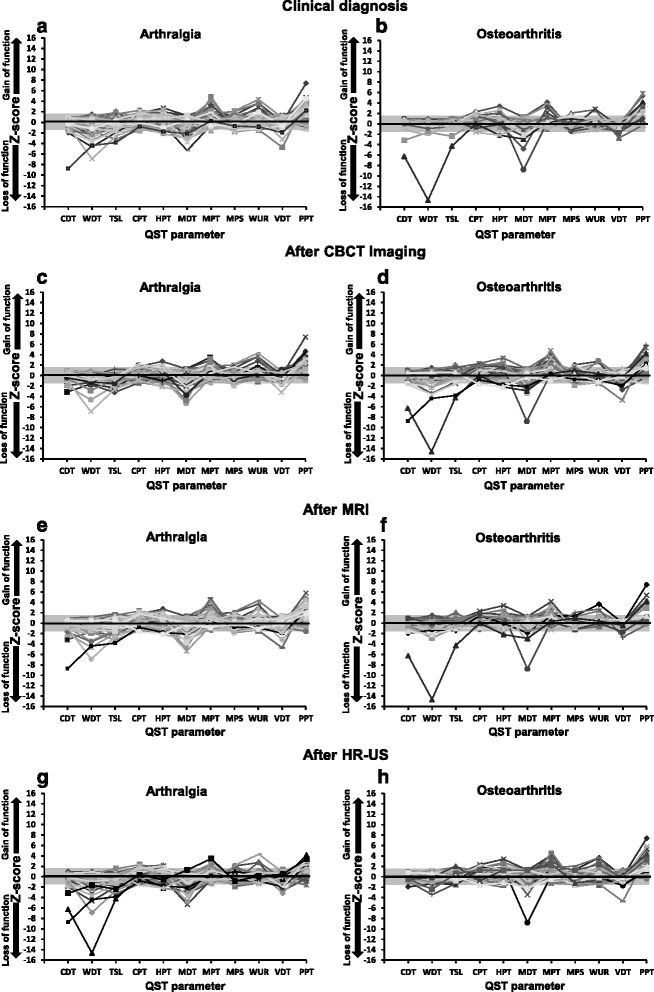
Somatosensory Z-score profiles of TMJ arthralgia and osteoarthritis patients at the test/most painful site diagnosed after clinical examination **a** arthralgia patients (*n* = 43) and **b** osteoarthritis patients (*n* = 15), after subsequent CBCT imaging **c** arthralgia patients (*n* = 28) and **d** osteoarthritis patients (*n* = 30), following MRI **e** arthralgia patients (*n* = 44) and **f** osteoarthritis patients (*n* = 14), and finally characterized based on HR-US **g** arthralgia patients (*n* = 28) and **h** osteoarthritis patients (*n* = 30). The grey zone indicates a Z-score between−1.96 and 1.96, representing the normal range of healthy subjects. A score above 1.96 indicates a gain in somatosensory function and a score below−1.96 indicates loss of somatosensory function. TMJ, temporomandibular joint; QST, quantitative sensory testing; CDT, cold detection threshold; WDT, warm detection threshold; TSL, thermal sensory limen; CPT, cold pain threshold; HPT, heat pain threshold; MDT, mechanical detection threshold; MPT, mechanical pain threshold; MPS, mechanical pain sensitivity; WUR, wind-up ratio; VDT, vibration detection threshold; PPT, pressure pain threshold, CBCT, cone beam computerized tomography; MRI, magnetic resonance imaging, HR-US, high resolution ultrasonography

The most frequent somatosensory abnormalities at the test site with regards to somatosensory gain of function were PPT and MPT in pure arthralgia patients and PPT, MPT and HPT in OA patients diagnosed as OA on combined examinations, and with regards to somatosensory loss of function were MDT and TSL in pure arthralgia patients and MDT and WDT in OA patients diagnosed as OA on combined examinations (Table 1). The QST sensory profiles of the arthralgia and OA patients at the test site of the TMJ, shown as Z-scores, for each examination modality are illustrated in Fig. 3 and for combined examinations are illustrated in Additional file 3: Figure S2.

Somatosensory abnormalities were also detected at the control site or non/less painful side of TMJ of arthralgia and OA patients to a lesser degree. The frequency of patients showing absolute abnormalities at the test site and control site for each and combined examination modalities is shown in Table 2. The most frequent somatosensory abnormalities at the control site of TMJ in arthralgia and OA patients, diagnosed regardless of examination modalities used were in terms of gain of function were PPT, WUR and MPT, and PPT and MPT, respectively. The most frequent abnormalities indicating loss of function at the control site of arthralgia and OA patients were MDT and WDT, and MDT and CDT, respectively. There was no occurrence of PHS or DMA at any of the sites. As expected, a few somatosensory abnormalities were also detected in the healthy controls, with a total of 31.7 % showing 1 or more values outside the 95 % CI (Table 1) [15, 42].

**Table 2:** Frequency (%) of TMJ arthralgia and osteoarthritis patients diagnosed after each and combined examination modalities, showing Z-score values outside the reference 95 % confidence interval of the reference data at test and control site

	Arthralgia patients		Osteoarthritis patients	
	Test site	Control site	Test site	Control site
	*n* (%)	*n* (%)	*n* (%)	*n* (%)
Clinical diagnosis	38 (88.4)	31 (72.1)	14 (93.3)	12 (80.0)
CBCT	25 (89.3)	21 (75.0)	27 (90.0)	21 (70.0)
MRI	38 (86.4)	31 (70.5)	14 (100.0)	12 (85.7)
HR-US	27 (96.4)	23 (82.1)	25 (83.3)	20 (66.7)
Combined	15 (100.0)	13 (86.7)	9 (100.0)	7 (77.7)

*Abbreviations*: *TMJ* temporomandibular joint, *CBCT* Cone beam computed tomography, *MRI* Magnetic resonance imaging, *HR-US* high resolution ultrasonography

Mechanical hyperalgesia without sensory loss (L0G2) was the most frequent coding in both OA and arthralgia patients diagnosed in accordance with each and combined examination modalities except for clinically diagnosed OA patients, where hypoesthesia to mechanical tactile stimuli along with mechanical hyperalgesia (L2G2) was the leading combination. For detailed results, see online resource 1. The distribution of the participants in each group according to the LossGain coding system consisting of both absolute (abnormal Z-scores) and relative (abnormal side-to-side difference) abnormalities for each and combined examination modalities is presented in Additional file 2: Table S2.

The ANOVA of QST data for each examination method demonstrated differences comparing arthralgia and OA patient groups and controls for MPT and PPT, with patients being more sensitive to pressure pain and pin-prick stimuli compared to controls (*P* < 0.005) (Table 3 and Additional file 2: Table S3). Further, for combined examinations, both pure arthralgia and OA patients diagnosed as OA on all three imaging modalities demonstrated increased sensitivity to pressure pain compared to controls (*P* < 0.001) (Table 3 and Additional file 2: Table S3).

**Table 3:** ANOVA comparing absolute values of each QST parameter across groups (TMJ arthralgia and osteoarthritis patients and healthy controls) at test and control site

	CDT	WDT	TSL	CPT	HPT	MDT	MPT	MPS	WUR	VDT	PPT
	*P* value	*P* value	*P* value	*P* value	*P* value	*P* value	*P* value	*P* value	*P* value	*P* value	*P* value
Clinical diagnosis^a^											
Group	ns	ns	ns	ns	ns	ns	0.005	ns	ns	ns	<0.001
Site	ns	0.021	ns	0.010	ns	0.001	ns	ns	0.047	<0.001	<0.001
Group X site	ns	ns	ns	ns	ns	0.018	ns	ns	_˜_ 0.051	ns	0.009
CBCT^a^											
Group	ns	ns	ns	ns	ns	ns	0.003	ns	0.035	ns	<0.001
Site	ns	0.025	ns	0.003	ns	0.006	ns	ns	ns	<0.001	<0.001
Group X site	ns	ns	ns	ns	ns	_˜_ 0.091	ns	ns	ns	ns	0.009
MRI^a^											
Group	ns	ns	ns	ns	ns	ns	0.001	ns	ns	ns	<0.001
Site	ns	0.010	ns	_˜_ 0.055	ns	<0.001	ns	ns	ns	<0.001	<0.001
Group X site	ns	ns	ns	ns	ns	0.015	ns	ns	ns	ns	0.010
HR-US^a^											
Group	0.023	0.005	0.002	ns	ns	0.022	0.003	ns	ns	ns	<0.001
Site	ns	0.025	ns	0.003	ns	0.006	ns	ns	ns	<0.001	<0.001
Group X site	ns	ns	ns	ns	ns	_˜_ 0.086	ns	ns	ns	0.017	0.007
Combined^a^											
Group	ns	0.010	<0.001	ns	0.043	_˜_ 0.058	<0.001	n s	ns	ns	<0.001
Site	ns	ns	ns	0.046	ns	0.008	ns	ns	ns	_˜_ 0.062	0.015
Group X site	ns	ns	ns	ns	ns	_˜_ 0.053	ns	ns	ns	0.003	_˜_ 0.067

*Abbreviations*: *ANOVA* analysis of variance, *TMJ* temporomandibular joint, *QST* quantitative sensory testing, *CBCT* Cone beam computed tomography, *MRI* Magnetic resonance imaging, *HR-US* high resolution ultrasonography, *CDT* cold detection threshold (°C), *WDT* warm detection threshold (°C), *TSL* thermal sensory limen (°C), *CPT* cold pain threshold (°C), *HPT* heat pain threshold (°C), *MDT* mechanical detection threshold (mN), *MPT* mechanical pain threshold (mN), *MPS* mechanical pain sensitivity (mean pain rating, 0–100), *WUR* windup ratio (ratio of pain rating), *VDT* vibration detection threshold (/8), *PPT* pressure pain threshold (kPa), ns not significant, _*˜*_ tendency towards a significant effect

^a^Patients were classified into TMJ arthralgia and osteoarthritis based on the diagnosis made after each examination modality (i.e., clinical examination, CBCT, MRI and HR-US and combined examinations (i.e., based on the presence or absence of degenerative changes on all imaging modalities at TMJ)). After which, absolute values of each QST parameter were compared between the groups. *P* values < .05 were considered significant

Additionally, ANOVA for side-to-side comparison for each examination modality revealed differences for the parameters WDT, CPT (except for MRI examination), MDT, VDT and PPT (Table 3 and Additional file 2: Table S3). (For details, see Additional file 1).

The ANOVA of QST data also showed a significant interaction between group and site for PPT, regardless of the examination methods applied for diagnosing arthralgia and OA patients (Table 3). The post-hoc tests revealed that the patient groups showed increased sensitivity to pressure pain compared with controls at both test (*P* < 0.001) and control site (*P* < 0.001). (For details, see Additional file 1).

### QST differences between TMJ arthralgia and OA patients

Based on clinical examination alone, there was a significant difference between the arthralgia and OA patient groups only for the parameter MDT, with OA patients being less sensitive to tactile stimuli compared with arthralgia patients (*P* = 0.026) and controls (*P* < 0.001) at the test site. Further, for patients diagnosed clinically in combination with CBCT, there was a tendency towards a significant difference with arthralgia patients being more sensitive to temporal summation of pain (WUR) compared with OA patients (*P* = 0.055). For the patients diagnosed after HR-US, arthralgia patients were less sensitive to warmth (*P* < 0.027), TSL (*P* < 0.006) and tactile stimuli (*P* < 0.048) compared with OA patients and healthy controls. Also, arthralgia patients were less sensitive to cold than OA patients (*P* = 0.027). OA patients showed increased sensitivity to pinprick stimuli compared with controls (*P* = 0.002), but not compared with arthralgia patients (*P* = 0.147). Interestingly, for MRI and HR-US diagnosed patients, OA patients showed increased sensitivity to pressure pain compared with arthralgia patients at both test (*P* < 0.025) and control site (*P* < 0.010). Further, pure arthralgia patients were less sensitive to warmth and TSL compared with controls (*P* < 0.008), whereas OA patients diagnosed based on combined modalities had somatosensory gain with regards to TSL compared with controls (*P* = 0.012). OA patients diagnosed based on combined modalities showed increased sensitivity to painful heat stimuli (*P* = 0.033) and pinprick stimuli (*P* < 0.001) compared with controls but not arthralgia patients. (Table 3 and Additional file 2: Table S3).

Comparison of QST data between the non-painful control site and the corresponding painful test site showed that the test site was more sensitive to pressure pain than the control site (*P* < 0.001; TS: 110.7 ± 7.6 kPa; CS: 133.0 ± 6.9 kPa). There was a tendency towards a significant difference for the parameters WDT (*P* = 0.071), MDT (*P* = 0.079) and VDT (*P* = 0.064).

### Conditioned pain modulation

Out of 58 patients, four declined to participate in the CPM experiment. Therefore, CPM results presented are based on 54 patients. The CPM results for each diagnostic level (clinical examination alone, clinical examination combined with CBCT, MRI and HR-US) are presented in Table 4. For each and combined examinations, ANOVA for the absolute PPT values showed that there were significant effects of group (*F* > 11.663; *P* < 0.001), site (*F* > 246.764; *P* < 0.001), session (*F* > 26.348; *P* < 0.001) and time (*F* > 20.332; *P* < 0.001). The post hoc tests revealed that the arthralgia and OA patients showed significantly lower PPT values compared with the healthy controls (*P* < 0.008). The PPT values at the thenar were significantly higher than at the TMJ (*P* < 0.001). The PPT values were significantly higher during the ice water session than during the neutral water session (*P* < 0.001). There was a significant increase in the PPT values during the leg immersion compared with before and after the leg immersion (*P* < 0.001).

**Table 4:** ANOVA comparing PPT values at most painful TMJ and thenar site across groups (healthy controls and TMJ arthralgia and osteoarthritis patients) during neutral water and ice water session

	Clinical diagnosis^a^	CBCT^a^	MRI^a^	HR-US^a^	Combined^a^
Factors	*P* value	*P* value	*P* value	*P* value	*P* value
Group	<0.001	<0.001	<0.001	<0.001	<0.001
Site	<0.001	<0.001	<0.001	<0.001	<0.001
Session	<0.001	<0.001	<0.001	<0.001	<0.001
Time	<0.001	<0.001	<0.001	<0.001	<0.001
Group X site	ns	ns	0.043	_˜_ 0.051	0.035
Group X session	0.018	0.020	0.020	0.018	ns
Group X time	<0.001	<0.001	<0.001	<0.001	0.003
Site X session	0.002	0.002	0.002	0.001	ns
Site X time	0.0215	0.002	ns	0.002	ns
Session X time	<0.001	<0.001	<0.001	<0.001	<0.001
Group X site X session	ns	ns	ns	ns	ns
Group X site X time	ns	ns	ns	ns	ns
Group X session X time	_˜_ 0.074	0.030	ns	ns	ns
Site X session X time	0.013	0.004	0.014	0.003	ns
Group X site X session X time	ns	ns	ns	ns	ns

*Abbreviations*: *ANOVA* analysis of variance, *TMJ* temporomandibular joint, *PPT* pressure pain threshold, *CBCT* Cone beam computed tomography, *MRI* Magnetic resonance imaging, *HR-US* high resolution ultrasonography, *ns* not significant, _*˜*_ tendency towards a significant effect

^a^Patients were classified into TMJ arthralgia and osteoarthritis based on the diagnosis made after each examination modality (i.e., clinical examination, CBCT, MRI and HR-US and combined examinations (i.e., based on the presence or absence of degenerative changes on all imaging modalities at TMJ)). After which they were compared between the groups. *P* values < .05 were considered significant

**Fig. 4:**
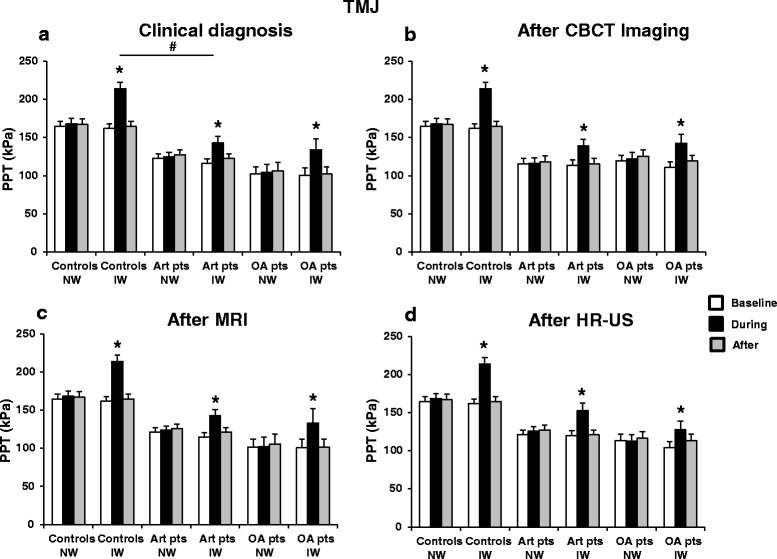
Pressure pain threshold (PPT) values (mean ± SEM) at the TMJ before (baseline), during and after the application of ice and neutral water in the healthy controls, TMJ arthralgia and osteoarthritis patients diagnosed based on the findings from **a** clinical examination **b** CBCT imaging **c** MRI and **d** HR-US.* indicates significantly different from the baseline, *P* < 0.001. # indicates significant difference between healthy controls and arthralgia patients, *P* = 0.046. TMJ, temporomandibular joint; CBCT, cone beam computerized tomography; MRI, magnetic resonance imaging; HR-US, high resolution ultrasonography; NW, neutral water; IW, ice water; Art pts, arthralgia patients; OA pts, osteoarthritis patients

**Fig. 5:**
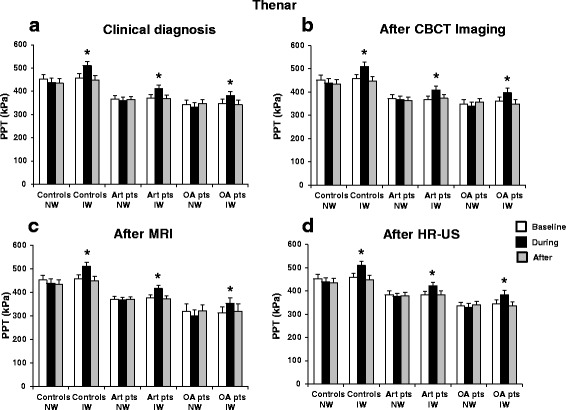
Pressure pain threshold (PPT) values (mean ± SEM) at thenar before (baseline), during and after the application of ice and neutral water in the healthy controls, TMJ arthralgia and osteoarthritis patients diagnosed based on the findings from **a** clinical examination **b** CBCT imaging **c** MRI and **d** HR-US. * indicates significantly different from the baseline, *P* < 0.001. TMD, temporomandibular disorders; TMJ, temporomandibular joint; CBCT, cone beam computerized tomography; MRI, magnetic resonance imaging; HR-US, high resolution ultrasonography; NW, neutral water; IW, ice water; Art pts, arthralgia patients; OA pts, osteoarthritis patients

There were many significant interactions between the factors (Table 4). The main findings were that both arthralgia and OA patients had lower PPT values than healthy controls during ice and neutral water sessions (*P* < 0.001) and also at the baseline, during and after the leg immersion (*P* < 0.001), indicating an overall increased sensitivity to pressure pain in the patient group. Interestingly, OA patients had lower PPTs during both sessions (*P* < 0.001) and at all time points (*P* < 0.001) compared with arthralgia patients based on all diagnostic modalities except for CBCT (*P* > 0.324). Also, an interaction between site, session and time revealed that there was a significant increase in PPT values during the leg immersion compared with before and after leg immersion during the ice water session at both the TMJ and thenar (*P* < 0.001) but not during neutral water session at the TMJ (*P* > 0.999) and thenar (*P* > 0.058) (Figs. 4 and 5). For combined diagnostic examinations, OA patients had lower PPT values at thenar compared with pure arthralgia patients (*P* = 0.029) (For detailed results, see Additional file 1, Additional file 3: Figure S3).

Further, to assess the CPM effect directly, the relative PPT changes from the baseline were compared between the arthralgia, OA patients and healthy controls for all the examination levels. For clinical examination alone, ANOVA showed a significant effect of subject group (*P* = 0.046) at TMJ with the post-hoc test revealing a decreased CPM effect in arthralgia patients (21.6 ± 3.8 %) compared with healthy controls (33.1 ± 2.9 %) (*P* = 0.047). However, there was no significant difference between the two patient groups (*P* = 0.263). For HR-US, there was a tendency towards a significant difference for OA patients showing reduced CPM effect compared with controls at the TMJ (*P* = 0.059). Further, for CBCT, MRI, and combined examinations, there was no significant difference in the relative PPT changes between the subject group neither at the TMJ (*P* > 0.116) nor at the thenar (*P* > 0.423) (Table 5).

**Table 5:** Relative PPT changes during the application of noxious conditioning cold stimulus in healthy controls and patient groups diagnosed after each and combined examination modalities

	TMJ				Thenar			
	Controls (%)	Arthralgia (%)	OA (%)	*P* value	Controls (%)	Arthralgia (%)	OA (%)	*P* value
Clinical diagnosis	33.1 ± 2.9	21.6 ± 3.8	32.1 ± 6.1	0.046*	13.8 ± 2.9	11.0 ± 1.7	11.9 ± 3.6	ns
CBCT	33.1 ± 2.9	22.9 ± 2.6	25.9 ± 5.9	ns	13.8 ± 2.9	11.5 ± 1.4	11.0 ± 2.7	ns
MRI	33.1 ± 2.9	23.2 ± 3.5	28.8 ± 8.6	ns	13.8 ± 2.9	10.7 ± 1.7	13.6 ± 3.7	ns
HR-US	33.1 ± 2.9	27.8 ± 4.1	20.7 ± 4.9	_˜_ 0.074	13.8 ± 2.9	10.8 ± 1.8	11.8 ± 2.5	ns
Combined	33.1 ± 2.9	21.0 ± 3.6	32.4 ± 15.0	ns	13.8 ± 2.9	9.0 ± 1.8	19.1 ± 6.1	ns

Data are given as mean values (%) ± standard errors of mean

*Abbreviations*: *PPT* pressure pain threshold, *TMJ* temporomandibular joint, *OA* osteoarthritis, *CBCT* Cone beam computed tomography, *MRI* Magnetic resonance imaging, *HR-US* high resolution ultrasonography, *ns* not significant, _*˜*_ tendency towards a significant difference

*P < 0.05 (*t*-test)

The NRS pain and unpleasantness scores were significantly higher for the ice water session (patients: pain: 69.0 ± 3.5, unpleasantness: 78.0 ± 2.9; healthy controls: pain: 61.3 ± 4.3, unpleasantness: 69.9 ± 4.3) than the neutral water (patients: pain: 0.2 ± 0.2, unpleasantness: 0.2 ± 0.2; healthy controls: pain: 0 ± 0, unpleasantness: 1.1 ± 0.6) in both patients and healthy controls (*P* < 0.001).

## Discussion

This is the first study to compare the somatosensory function at TMJ between TMJ arthralgia and OA patients using the full battery of 13 standardized QST parameters [17]. Also this is the first study to assess the function of the endogenous analgesic system in TMJ arthralgia and OA patients separately in comparison to age- and gender-matched healthy controls. Moreover, the patients were carefully characterized based on the findings from clinical examination and three different imaging techniques. The main findings were that both TMJ arthralgia and OA patients were associated with a higher frequency of somatosensory abnormalities than in healthy controls. Interestingly, a few, but significant differences in the QST parameters between the two groups of patients were demonstrated. The CPM effect was similar in patients and controls.

### Imaging

In this study, three different types of imaging techniques were employed - CBCT, MRI and HR-US in addition to a standardized clinical examination using the RDC/TMD. The patients were classified repeatedly into arthralgia or OA based on the findings from the different diagnostic modalities. It is interesting to see how the diagnosis changed at each level of examination. Moreover, diagnostic radiology is part of a larger system whose goal is to treat patients effectively and efficiently [46]. TMJ OA affects the cartilage, subchondral bone, synovial membrane, and other hard and soft tissues causing changes such as articular cartilage abrasion and deterioration, and thickening and remodeling of underlying bone [47, 48]. Therefore clinical criteria alone, without recourse to imaging are inadequate for valid and accurate diagnosis of OA [49]. Hence, patients with pain and coarse crepitus at the TMJ on clinical examination, but without degenerative changes on CBCT were grouped as arthralgia patients (Fig. 2). A few studies have considered flattening of the articulating surface of the condyle, fossa or eminence as related to OA [50]. Flattening was also observed in many patients in this study, however, it is viewed as a sign of remodeling and is considered as an indeterminate for OA [31]. Unlike knee OA, narrowed joint space in TMJ is not considered as a reliable indicator of TMJ OA, as joint space can also vary with disc displacements, with OA, and in normal joints during mastication [31, 51]. With regards to the disc morphology, modified disc shape is considered as an important feature of internal derangements of TMJ [52]. On the other hand, dynamic variations of the disc dimension suggest that the disk morphology is strongly related to the mandibular biomechanics [34]. Also, the relationship of fluid effusion to pain and OA is not yet clear [53, 54] and is considered as a sign that may appear before osteoarthritic changes occur [31]. Therefore, in the present study it was not considered as a sign of OA. At the time of devising this study, no standardized and valid criteria for the diagnosis of TMJ OA on HR-US was found. Moreover, for synovial hypertrophy which is considered as a sign of OA, no standard normal values of the width of the TMJ synovium were found. Therefore, we transformed the measurements of TMJ synovium of patients data into standardized Z-scores using healthy reference values. Z-score is a statistical measurement of a score’s relationship to the mean in a group of scores, where each individual’s measurement is related to the age- and gender-specific reference range and is displayed as the number of standard deviations above the normal mean. We employed one-tailed Z-scores, as the abnormal values/scores were considered always to be higher than the healthy reference data.

### Somatosensory function

Our previous study demonstrated that the majority of the TMD arthrogenous pain patients presented with at least one or more somatosensory abnormalities [15]. However, in that study, somatosensory function was not assessed separately in TMJ arthralgia and OA patients, due to a small sample size. In the present study, it was shown that more than 86 % of arthralgia patients and more than 83 % of OA patients diagnosed after each and combined examination techniques presented with at least one or more somatosensory abnormalities compared to an age- and gender- matched reference group (Table 2). In addition to the detailed somatosensory assessment, this study also reported on the direction (increased/decreased) of somatosensory abnormalities unlike other studies of TMJ pain origin [24, 55]. Regardless of the examination technique used, both groups of patients demonstrated increased sensitivity to pressure pain stimuli as the most frequently occurring somatosensory abnormality with regards to gain of function at the test site. This increased sensitivity is thought to be due to peripheral sensitization to mechanical stimuli of the articular tissues or the tissues overlying the TMJ, central sensitization, or both [56, 57]. This finding was in line with previous studies, where TMJ arthralgia, knee OA, myofascial TMDs and other musculoskeletal pain disorder patients have shown pressure pain hyperalgesia [22, 58–60]. As in our previous study [15], at the test site, pinprick hyperalgesia and pronounced temporal summation (WUR) were also frequently demonstrated by both groups of patients indicating central nociceptive sensitization. Such findings have also been previously reported in myofascial and arthrogenous TMD patients [59, 61, 62]. These findings were also evident on group analysis of PPT and MPT parameters, where both arthralgia and OA patients were more sensitive to painful pressure and pinprick stimuli compared with healthy controls. Interestingly, heat hyperalgesia was also commonly seen in both groups of patients. Such a finding has not been reported earlier in the arthrogenous TMD patients and in our previous study [15] it was seen only in a very few patients. Peripheral sensitization of C fibers may be responsible for heat hyperalgesia [19].

In terms of sensory loss of function, tactile hypoesthesia was the most commonly occurring somatosensory abnormality at the test site in both diagnostic subgroups of patients regardless of the examination techniques used. Reduced tactile sensation may be due to inhibitory mechanisms [63]. Similar findings were reported in studies conducted in knee OA [60]. However, a couple of studies employing different stimulus modality and response measures demonstrated tactile hyperesthesia in TMJ arthralgia patients [59, 64], indicating that the results may differ based on the study design.

Interestingly, somatosensory abnormalities were also detected at the control or less/non-painful TMJ to a lesser degree in both groups of patients diagnosed based on each individual and combined examination modalities (Table 2). At the control site, 70–87 % of arthralgia patients and 66–86 % of OA patients diagnosed after each individual and combined examination modalities demonstrated somatosensory abnormalities. However, only 40 % of the total group of patients had pain bilaterally at the joint and 60 % had pain only at one side, thus indicating generalized sensitivity suggestive of central sensitization in these patients [65]. This finding is in accordance with many other studies on persons with TMD pain, knee OA and other musculoskeletal pain disorders [22, 58–60, 66].

The most frequent LossGain score encountered in both arthralgia and OA patients was L0G2 (no somatosensory loss combined with gain of mechanical somatosensory function). Further, looking at the individual QST parameters, somatosensory gain with regard to PPT and MPT, ie, mechanical hyperalgesia, were the most frequently encountered abnormalities in both group of patients which corresponds with the LossGain score L0G2. L0G2 was also seen as the most frequently occuring score in patients with trigeminal neuralgia and atypical odontalgia in studies by Maier et al. [19] and Baad-Hansen et al. [42], respectively. LossGain scoring employs both absolute and relative abnormalities. Inclusion of both absolute and relative abnormalities provide high specificity and increased diagnostic sensitivity [42]. However, incorporating the side-to-side differences (relative abnormalities) in the evaluation of cases with bilateral somatosensory abnormalities presents further difficulties [19, 42]. This is one of the disadvantages of using LossGain coding. However, in the present study, LossGain coding was applied only to see the combination of abnormalities displayed by the patients and no analysis was performed to differentiate between the groups.

A few somatosensory deviations, ie, Z-scores oustside the 95 % CI, were also detected in the healthy reference group. A total of 31.7 % of healthy controls showed one or more values outside the 95 % CI. However, based on the simple calculation of chance probability of being healthy and having at least 1 of the 11 values outside the 95 % CI ([1–0.95^11^] = 43.1 %), this frequency is actually lower than would be expected [19]. Similar findings were also seen in other studies assessing somatosensory function [15, 19, 42]. This high probablility of healthy controls showing at least one abnormal value may be considered as a drawback to the very comprehensive QST protocol [19, 42].

### QST differences between TMJ arthralgia and OA patients

Though the psychophysical responses given by both groups of TMD pain patients for each QST parameter appeared similar, a few, but significant differences in the somatosensory function between the patient groups also existed. Interestingly, TMJ OA patients were mostly associated with somatosensory gain of function (hyperalgesia) when compared with arthralgia patients, whereas arthralgia patients demonstrated a higher frequency of negative somatosensory signs compared with OA patients. Hyperalgesia was documented in OA, where patients showed increased sensitivity to blunt pressure at both test and control sites compared with arthralgia patients and also OA patients diagnosed on combined examinations showed hyperalgesia to heat pain and punctuate stimuli compared with controls but not in comparison with so-called pure arthralgia patients. Hyperalgesia to pressure pain, pinprick stimuli and heat pain has also been shown in patients with OA of other joints like knee and hand [60, 67]. It is thought that this heightened pain sensitivity results from both central as well as peripheral sensitization [41, 65]. Moreover, the fact that differentiates between the two TMJ pain conditions is the presence of degenerative changes. It has been demonstrated that degenerative joint articular cartilage changes in symptomatic TMJs are almost always associated with an inflammatory component that is reflected as synovitis [68]. In OA, inflammation of the TMJ results in increased release of several pro-inflammatory cytokines, in particular tumour necrosis factor-α (TNFα) and interleukins (1,6,12 and 17), which have been found to mediate cartilage destruction and joint remodeling [56, 69]. Further, significant correlations have been found between increased proinflammatory cytokine levels such as IL-1β and PPT suggesting a biological association between IL-1β and hyperalgesia in the TMJ region [70, 71]. Also, to some degrees, inflammation can activate mechanically sensitive nociceptors within the joint by increasing intraarticular pressure, further contributing to joint-related pain [72]. As in studies with knee OA patients, along with hyperalgesia, TMJ OA patients also showed reduced tactile sensitivity compared with arthralgia patients. This pain-related hypoesthesia might be due to activation of descending inhibitory systems [60]. Arthralgia patients demonstrated reduced sensitivity to thermal (CDT, WDT and TSL) and mechanical (MDT) non-noniceptive parameters compared with OA patients and controls. The process underlying the hypoesthetic changes in musculoskeletal conditions are unclear [73] however, it has been proposed to occur as a result of inhibitory processes [63, 73, 74]. Previous studies evaluating thermal function in TMD patients have mostly assessed only thermal pain thresholds [23, 75] and only relatively few studies have assessed thermal detection thresholds in myofascial TMD and arthralgia patients, where no changes for the sensitivity of CDT and/or WDT have been reported [21, 64]. Further, use of different devices, testing algorithms, and settings like variations in thermode size, baseline temperature and stimulus duration may vary the results [76]. In the present study, decreased sensitivity to tactile stimuli was also shown by arthralgia patients. This finding was in contrast to two other studies where arthralgia patients showed decreased MDT (increased sensitivity) [59, 64]. However, these studies used electrical stimuli to measure Aβ fiber function. Thus, these subtle differences in QST between TMJ arthralgia and OA patients may shed further light on the pathophysiological mechanisms underlying pain in these conditions. Further, no significant difference in the distribution of myofascial pain diagnoses across the arthralgia and OA patients indicated that the comorbid diagnoses of myofascial pain did not have any major influence on the somatosensory function in these patient groups. Further studies with a large group I distribution in one of the TMJ pain patient groups will be needed to further evaluate the effect of myofascial pain on somatosensory function.

### Conditioned pain modulation

Impairment of the endogenous analgesic systems has been implicated as a contributing factor in the development and maintenance of many chronic pain conditions including TMD [77]. However, in the present study, the two TMJ pain conditions—TMJ arthralgia and OA-were not associated with diminished endogenous pain inhibition as measured by CPM. This finding was in line with two recent CPM studies in TMD pain patients where no significant difference in CPM was found between the patients and controls [15, 30]. One of these studies showed reduced temporal summation of pain on application of painful cold to the hand [30] and another study demonstrated decreased sensitivity to blunt pressure on application of noxious cold to the foot [15]. Similarly in the present study, increased PPTs at both the painful (TMJ) and control (thenar) sites on application of painful cold stimulus to the foot was seen in both TMJ arthralgia and OA patients diagnosed after each individual and combined examinations. Moreover, no significant differences in the CPM effect (relative changes in PPT) between controls, arthralgia and OA patients were seen except for clinically diagnosed arthralgia patients showing less CPM at the TMJ compared to controls. Though the CPM effect was less in these arthralgia patients compared with controls, it was still significant. Interestingly, OA patients displayed lower PPT values during both ice and neutral water session and at all time points compared to arthralgia patients further supporting the finding from QST that these patients show more pronounced hyperalgesia to pressure pain than arthralgia patients suggestive of central changes. In contrast to the findings of this study, a few CPM studies in TMD and OA patients have shown impaired CPM effects indicative of dysfunctional endogenous analgesic systems [27, 29]. At present, the reason for the inconsistency between the findings of CPM in TMD patients cannot be found. A possible explanation could be that the capacity of pain modulation can be expressed differently in response to the application of different CPM paradigms [78]. More studies using a standardized CPM protocol are required to clarify the issue of functioning of endogenous analgesic systems in TMD pain patients. Nevertheless, the results from the present study indicate that the CPM effect was intact at both the segmental and extrasegmental sites in both group of TMJ pain patients diagnosed after each and combined examinations. Thus suggesting that the TMJ pain in these patients may not be associated with a compromised endogenous pain inhibitory systems.

## Conclusions

The assessment of sensory and pain thresholds using a standardized QST protocol uncovered an array of somatosensory abnormalities in both TMJ arthralgia and OA patients diagnosed clinically and based on different imaging modalities. Profiles of somatosensory function were able to differentiate between these conditions. TMJ OA patients demonstrated more pronounced hyperalgesia compared with arthralgia patients suggestive of central as well as peripheral sensitization. In contrast, arthralgia patients were mostly associated with somatosensory loss of function to non-nociceptive parameters compared with OA patients. Thus, our study points towards involvement of different underlying pain mechanisms in different TMJ pain conditions, which may have significant impact on diagnosis and rational management of these disorders. The CPM effects were similar in both patient groups and healthy controls, implying that chronic painful TMJ may not be associated with dysfunctional endogenous analgesic systems. Now, it remains to be tested if subgroups of TMD pain patients with different underlying pain mechanisms respond to different therapeutic approaches.

## Additional files

Additional file 1: Supplementary material (DOCX 37 kb)

Additional file 2: Supplementary Tables (DOCX 26 kb)

Additional file 3: Supplementary Figures (PPTX 2246 kb)
