# Supplementary material for: Pain profiling of patients with temporomandibular joint arthralgia and osteoarthritis diagnosed with different imaging techniques
Source: J Headache Pain. 2016 Jun 27;17(1):61. doi: 10.1186/s10194-016-0653-6 (PMC4923011; doi:10.1186/s10194-016-0653-6)
Supplement: Supplementary file 1 — Supplementary material (DOCX 37 kb) [file 10194_2016_653_MOESM1_ESM.docx]

**Supplementary material:**

**Pain profiling of patients with temporomandibular joint**

**arthralgia and osteoarthritis diagnosed with different imaging techniques**

Simple Futarmal Kothari^1,2^, Lene Baad-Hansen^1,2^, Lars Bolvig Hansen^3^, Niels Bang^3^,

Leif Hovgaard Sørensen^4^, Helle Wulf Eskildsen^4^, Peter Svensson^1,2,5^

^1^Section of Orofacial Pain and Jaw Function, Institute of Odontology and Oral Health, Aarhus University, Denmark

^2^Scandinavian Center for Orofacial Neurosciences (SCON)

^3^Department of Radiology, Aarhus University Hospital, Denmark

^4^Department of Neuroradiology, Aarhus University Hospital, Denmark

^5^Department of Dental Medicine, Karolinska Institutet, Huddinge, Sweden

**Corresponding author:**

Simple Futarmal Kothari, BDS, PhD student

Section of Orofacial Pain and Jaw Function

Institute of Odontology and Oral Health, Aarhus University

Vennelyst Boulevard 9

DK-8000, Aarhus C, Denmark.

Email: [simple.futarmal@odont.au.dk](mailto:simple.futarmal@odont.au.dk)

Phone number: +45 5039 5697

Fax number: +45 8942 4297

**Methods**

***Quantitative sensory testing***

*Thermal detection and pain thresholds and the number of paradoxical heat sensations.*

As a measure of Aδ-fiber function, thresholds for cold detection (CDT) and cold pain (CPT) were assessed. C-fiber function was tested by assessing thresholds for warmth detection (WDT). Heat pain thresholds (HPT) determined with contact thermal stimulators are considered to assess mainly Aδ-fiber function in skin. The presence of paradoxical heat sensations upon cold stimulation is usually considered indicative of a disturbance in Aδ-cold fiber function or of central sensitization [1, 2].

All thermal testing was performed using a PATHWAY thermal sensory testing device (MEDOC Ltd., Ramat Yishai, Israel) [3, 4]. The baseline temperature was 32ºC and the contact area of thermode was 30 mm x 30 mm (square surface) [3, 5]. The cut-off temperature for cold stimuli (CDT, CPT) was 0ºC, and for warm and hot stimuli (WDT, HPT) was 50ºC [3, 5]. CDT, WDT, paradoxical heat sensations by using the thermal sensory limen procedure (TSL), CPT and HPT were assessed using ramped stimuli of 1ºC/s [6]. For all thresholds, the mean of three threshold temperature measurements were calculated [1].

*Mechanical detection threshold*

To assess Aβ-fiber function, mechanical detection thresholds (MDT) were measured using a set of standardized von Frey filaments with rounded tips of 0.5 mm diameter (OptiHair_2_, MARSTOCK nervtest, Marburg, Germany) [1, 3]. The OptiHair_2_ set contains 12 monofilaments, which exerts forces between 0.25 and 512 mN. The monofilament was applied perpendicularly to the examination site. Contact time was 1-2 s [1]. Using the method of limits, five threshold determinations were made, each with a series of ascending and descending stimulus intensities [1]. The final threshold was the geometric mean of these five series [1].

*Mechanical pain threshold*

To assess Aδ-fiber function in relation to mechanical stimuli, a standardized set of seven custom-made weighted pinprick stimulators (made at Aarhus University, Aarhus, Denmark) with fixed stimulus intensities (8, 16, 32, 64, 128, 256 and 512 mN) and a flat contact surface of Ø 0.2 mm were used to determine the mechanical pain threshold (MPT) [3, 4]. All pinprick tests were made with the stimulator perpendicular to the examination site. The method of limits that was used to determine the MDT was also used to determine MPT [1].

*Stimulus-response-function: mechanical pain sensitivity for pinprick stimuli and dynamic mechanical allodynia.*

The sensitivity to sharp stimuli as mediated by Aδ-fibers and pain upon light stroking was measured. Two sets of instruments were used for this stimulus-response assessment. To determine mechanical pain sensitivity (MPS), seven custom-made weighted pinprick stimulators were used [3, 4]. The pinprick stimulators and the application technique were the same as those used to determine MPT. Three tactile stimulators were used to determine dynamic mechanical allodynia (DMA): *(i)* a cotton wisp that exerted a force of ~3mN, *(ii)* a cotton wool tip (Q-tip) attached to a flexible handle that exerted a force of ~100mN, and *(iii)* a standardized brush (Somedic, Sweden) that exerted a force of ~200 mN [1, 3, 4]. The tactile stimulator was applied in a single stroke over about 1–2 cm in length of skin. A series of 10 measurements was made 5 times, each with the 10 stimulators (7 pinprick and 3 tactile stimulators) applied in a different order, as specified in the DFNS protocol [1, 7]. For each of the resulting 50 stimuli, the subject chose a pain rating on a 0–100-point numerical rating scale (NRS) with the endpoints ‘0’ indicating “no pain” and ‘100’ indicating “most intense pain imaginable” [3, 4]. The MPS was calculated as the geometric mean of all numerical ratings for pinprick stimuli [3, 4]. DMA was calculated as the geometric mean of all numerical ratings for tactile stimuli [1].

*Wind-up ratio to repetitive pinprick stimuli*

To measure the perceptual correlate of temporal pain summation, the wind-up ratio (WUR) for repetitive pinprick stimuli was assessed. The perceived magnitude of a single pinprick stimulus was referenced to that of a train of 10 pinprick stimuli with the same force, repeated at a rate of 1 Hz and kept constant using a metronome (MA-30 Digital metronome, KORG®, Tokyo, Japan) [3-5]. A custom-made pinprick stimulator as mentioned above was used for WUR assessment [6]. The instrument that delivered a force, which the subject perceived as “slightly painful” was chosen for the tests and could vary from site to site and from individual to individual [4]. To determine which instrument the subject perceived to be slightly painful, the 128-mN stimulator was first tried [1]. If the response was 0 (not painful), the test was repeated with the next stimulator in the series that exerted a stronger force. If the subject perceived the stimulus as intolerable, the next stimulator in the series that exerted a weaker force was used [3, 4]. Otherwise, if the subject’s numerical response was >0, indicating pain, but not intolerable pain, the stimulator was selected for testing at that site. If a subject did not perceive the 512-mN stimulator to be painful, the test was abandoned [3, 4]. Five single pinprick stimuli were alternated with five trains of 10 stimuli. The mean pain rating of the trains was then divided by the mean pain rating for single stimuli to render five WURs that represented temporal pain summation for the site [6]. The mean value of the five WURs was used for analysis [1, 4].

*Vibration detection threshold*

One way of assessing Aβ-fiber function is to determine the vibration detection threshold (VDT) using a Rydel-Seiffer graded tuning fork (64 Hz, 8/8 scale) [1]. VDT represents the only disappearance threshold within the proposed QST battery [1]. The tuning fork was placed over a bony prominence and the subject indicated when the vibration could no longer be sensed [3, 5]. On the 9-point (0-8 out of 8) scale measuring intensity of vibration, values with a discrimination down to 0.5 were noted [7]. The mean of three trials was calculated [4].

*Pressure pain threshold*

The pressure pain threshold (PPT) is the final test in the protocol. It was measured to test deep pain sensitivity, which is probably mediated through C- or Aδ-fibers [6]. A digital pressure algometer (SOMEDIC AB, Sollentuna, Sweden) with a probe area of 1 cm^2^ was used [3, 5]. During the test, pressure was increased at a rate of 50 kPa/s [6]. At the first painful sensation, the subjects pressed a button to interrupt stimulation and the PPT was defined as the amount of pressure (kPa) at which the subject first perceived pain [8]. The PPT was determined as the mean of three recordings [1].

**Results**

*Patient description*

Out of 58 patients (116 joints), 23 patients (46 joints) had pain bilaterally and 35 patients (35 joints) had pain unilaterally at the TMJ. Therefore, to get a better picture of the number of joints involved, after clinical diagnosis, they were divided into 3 groups – healthy joints (35 joints), joints with arthralgia (63 joints) and joints diagnosed as having OA (18 joints). Joints diagnosed as arthralgia (63 joints) and OA (18 joints) were re-classified after CBCT imaging into one of the two diagnoses depending on the presence or absence of degenerative changes. Therefore, joints diagnosed as OA had arthralgia, coarse crepitus and degenerative changes. Further, these joints were again classified into arthralgia and OA based on the findings from MRI and HR-US. The healthy non-painful joints (35 joints) were classified after CBCT imaging into normal joints and joints diagnosed as having osteoarthrosis. Joints with osteoarthrosis show degenerative changes but without pain. The healthy joints were further classified as being normal or showing osteoarthrosis after MRI and HR-US examinations. On MRI, joints were also classified as having disc displacements. A flowchart showing the classification of joints into different diagnosis after clinical examination and imaging techniques have been presented in supplementay Fig. 1.

Clinical diagnosis plus CBCT as a reference, HR-US had sensitivity of 60.0% and specificity of 63.2% for the diagnosis of osteoarthritis. The sensitivity and specificity of MRI for the diagnosis of OA was not calculated as none of the healthy controls underwent MRI of the TMJ.

*Quantitative sensory testing / Sensory abnormalities*

The distribution of the participants in each group according to the LossGain coding system consisting of both absolute (abnormal Z-scores) and relative (abnormal side-to-side difference) abnormalities for each and combined examination modalities is presented in supplementary Table S2. In OA patients, diagnosed based on the degenerative changes on MRI, hypoesthesia to thermal stimuli with mechanical hyperalgesia (L1G2) also occurred most frequently along with L0G2. Mechanical hypoesthesia with mixed hyperalgesia (L2G3) was also seen as most frequent occurring combination along with L0G2 in arthralgia patients, showing no degenerative changes on HR-US. More than 60% of the healthy reference controls did not show any abnormality (L0G0), whereas only one or two patients per patient group did not show any abnormality.

ANOVA for side-to-side comparison for each examination modality revealed differences for the parameters WDT, CPT (except for MRI examination), MDT, VDT and PPT (Table 3 and supplementary Table S3). Regardless of the examination modality used for diagnosing arthralgia and OA patients, in a side-to-side comparison, it was revealed that compared with the control site (CS), the test site (TS) was significantly less sensitive to warmth (*P* < 0.025), tactile stimuli (*P* < 0.006) and vibration (*P* < 0.001) and more sensitive to cold pain (*P* < 0.005) and pressure pain (*P* < 0.001). Further for combined examinations, side-to-side comparison showed differences for parameters MDT, CPT and PPT with test site being less sensitive to tactile stimuli (*P* = 0.001) and more sensitive to cold pain stimulus (*P* = 0.016) and pressure pain (*P* = 0.033) than control site (supplementary Table S3).

The ANOVA of QST data also showed interaction between group and site for VDT, only in patients diagnosed after HR-US. At control site, arthralgia patients were less sensitive to vibration compared with controls (*P* = 0.003) and OA patients (*P* < 0.001) but not at test site (*P* > 0.925). Also, for combined diagnosis, significant interaction between group and site was found only for VDT with pure arthralgia patients being less sensitive to vibration compared with controls (*P* < 0.001) and OA patients (*P* = 0.001) at control site but not at test site (*P* > 0.718). For MDT and PPT, there was a tendency towards a significant interaction between group and site (Table 3 and supplementary Table S3).

*Conditioned pain modulation*

For all the examinations, ANOVA for the absolute PPT values showed that there were significant interactions between subject group and session (*F* > 4.061; *P* < 0.020), subject group and time (*F* > 4.228; *P* < 0.002), site and session (*F* > 18.389; *P* < 0.001), session and time (*F* > 60.572; *P* < 0.001), site and time (*F* > 3.919; *P* < 0.021) except for MRI, and site, session and time (*F* > 4.311; *P* < 0.014) for all examinations. For MRI examination, there was a significant interaction between group and site (*F* = 3.239; *P* = 0.043). There was also a significant interaction between subject group, session and time for CBCT (*F* = 2.727; *P* = 0.030). The results of CPM for each examination technique are presented in Table 4. The site and session interaction showed that the PPT values at thenar were significantly higher than the TMJ during both the sessions (*P* < 0.001). The session and time interaction showed that there was significant increase in PPT values during the leg immersion compared with baseline during the ice water session (*P* < 0.001) but not during the neutral water session (*P* = 0.766). The site and time interaction showed that the PPT values were significantly higher at both sites during the leg immersion compared with before and after leg immersion (*P* < 0.001). Also PPT values were significantly higher at thenar during all time points compared with TMJ (*P* < 0.001). The subject group and site interaction for MRI examination showed that the PPT values were significantly lower at both sites in the patient groups compared with healthy controls (*P* < 0.022). The interaction between the subject group, session and time for CBCT showed that the arthralgia and OA patients exhibited significantly decreased PPT values at baseline, during and after the leg immersion during both the sessions compared with healthy controls (*P* < 0.001).

For combined examinations, ANOVA for the absolute PPT values showed that there were significant interactions between subject group and site (*F* = 3.531; *P* = 0.035), site and session (*F* = 9.292; *P* = 0.003), and session and time (*F* = 30.205; *P* < 0.001). The subject group and site interaction showed that both pure arthralgia and OA patients had lower PPTs than the controls at thenar (*P* < 0.004). Also OA patients had lower PPTs at thenar compared with pure arthralgia patients (*P* = 0.029). The site and session interaction showed that the PPT values at TMJ were significantly lower than at thenar during both the sessions (*P* < 0.001). The session and time interaction showed that the PPT values significantly increased during the leg immersion compared with baseline during the ice water session (*P* < 0.001) but not during the neutral water session (*P* = 0.521) (supplementary Fig. 3). (Table 4)

**Figure Legends**

**Supplementary Fig 1** A flowchart showing the classification of temporomandibular joints (TMJ) of 58 TMD pain patients into different diagnosis after RDC/TMD clinical examination and 3 different imaging techniques. The joints were divided into 3 groups - healthy joints, joints diagnosed as arthralgia and osteoarthritis (OA) based on clinical examination. Then, the joints grouped as arthralgia and OA on clinical examination were re-classified based on the presence or absence of degenerative changes on CBCT imaging irrespective of coarse crepitus at the joint as OA and arthralgia respectively. Further, these joints were diagnosed as having OA or arthralgia based on the presence or absence of the degenerative changes respectively on MRI and HR-US. All the joints diagnosed as arthralgia and OA have pain. In addition, concordance between MRI and HR-US for the diagnosis of arthralgia and OA is also presented, where “-” indicated no degenerative changes seen and “+” indicated degenerative changes found. Accordingly -MRI-US meant joints diagnosed as arthralgia and +MRI+US meant joints belonging to OA group. The healthy non-painful joints were divided into healthy joints and joints diagnosed as osteoarthrosis (OH) after CBCT imaging. Joints with OH have no pain. After which, these joints were further re-classified as normal joints (NJ), OH or disc displacements (DD) based on the findings on MRI and HR-US. RDC/TMD, Research Diagnostic Criteria for Temporomandibular disorders; TMJ, temporomandibular joint; CBCT, cone beam computerized tomography; MRI, magnetic resonance imaging; US, high resolution ultrasonography; HR-US, high resolution ultrasonography; HJ, healthy joint, * indicates metal artefact at one joint on MRI

**Supplementary Fig 2** Somatosensory Z-score profiles of TMJ pure arthralgia (n=15) and osteoarthritis patients (OA) (n=9) showing degenerative changes on all imaging modalities (cone beam computerized tomography, magnetic resonance imaging and high resolution ultrasonography at the test/most painful site. Grey zone indicates a Z-score between -1.96 and 1.96, representing the normal range of healthy subjects. A score above 1.96 indicates a gain in somatosensory function and a score below -1.96 indicates loss of somatosensory function. TMJ, temporomandibular joint; QST, quantitative sensory testing; CDT, cold detection threshold; WDT, warm detection threshold; TSL, thermal sensory limen; CPT, cold pain threshold; HPT, heat pain threshold; MDT, mechanical detection threshold; MPT, mechanical pain threshold; MPS, mechanical pain sensitivity; WUR, wind-up ratio; VDT, vibration detection threshold; PPT, pressure pain threshold

**Supplementary Fig 3** Pressure pain threshold (PPT) values (mean ± SEM) before (baseline), during and after the application of ice and neutral water at (a) TMJ and (b) thenar in the healthy controls, pure arthralgia patients and osteoarthritis patients (OA) showing degenerative changes on all imaging modalities (cone beam computerized tomography, magnetic resonance imaging and high resolution ultrasonography). * indicates significantly different from the baseline, *P* < 0.001. TMJ, temporomandibular joint; NW, neutral water; IW, ice water; Art pts, arthralgia patients; OA pts, osteoarthritis patients

**References**

1. Rolke R, Baron R, Maier C, Tolle TR, Treede RD, Beyer A, Binder A, Birbaumer N, Birklein F, Botefur IC, Braune S, Flor H, Huge V, Klug R, Landwehrmeyer GB, Magerl W, Maihofner C, Rolko C, Schaub C, Scherens A, Sprenger T, Valet M, Wasserka B (2006) Quantitative sensory testing in the German Research Network on Neuropathic Pain (DFNS): standardized protocol and reference values. Pain 123:231-243

2. Susser E, Sprecher E, Yarnitsky D (1999) Paradoxical heat sensation in healthy subjects: peripherally conducted by A delta or C fibres?. Brain 122 ( Pt 2):239-246

3. Kothari SF, Baad-Hansen L, Oono Y, Svensson P (2015) Somatosensory assessment and conditioned pain modulation in temporomandibular disorders pain patients. Pain 156:2545-2555

4. Baad-Hansen L, Pigg M, Ivanovic SE, Faris H, List T, Drangsholt M, Svensson P (2013) Intraoral somatosensory abnormalities in patients with atypical odontalgia--a controlled multicenter quantitative sensory testing study. Pain 154:1287-1294

5. Kothari SF, Baad-Hansen L, Andersen K, Svensson P (2014) Neurosensory assessment in patients with total reconstruction of the temporomandibular joint. Int J Oral Maxillofac Surg 43:1096-1103

6. Pigg M, Baad-Hansen L, Svensson P, Drangsholt M, List T (2010) Reliability of intraoral quantitative sensory testing (QST). Pain 148:220-226

7. Rolke R, Magerl W, Campbell KA, Schalber C, Caspari S, Birklein F, Treede RD (2006) Quantitative sensory testing: a comprehensive protocol for clinical trials. Eur J Pain 10:77-88

8. Kothari SF, Kothari M, Baad-Hansen L, Svensson P (2012) Comparison of techniques for evaluation of deep pain sensitivity in the craniofacial region. J Orofac Pain 26:225-232
