# Supplementary material for: Pain profiling of patients with temporomandibular joint arthralgia and osteoarthritis diagnosed with different imaging techniques
Source: J Headache Pain. 2016 Jun 27;17(1):61. doi: 10.1186/s10194-016-0653-6 (PMC4923011; doi:10.1186/s10194-016-0653-6)
Supplement: Supplementary file 2 — Supplementary Tables (DOCX 26 kb) [file 10194_2016_653_MOESM2_ESM.docx]

**Supplementary tables:**

**Pain profiling of patients with temporomandibular joint**

**arthralgia and osteoarthritis diagnosed with different imaging techniques**

Simple Futarmal Kothari^1,2^, Lene Baad-Hansen^1,2^, Lars Bolvig Hansen^3^, Niels Bang^3^,

Leif Hovgaard Sørensen^4^, Helle Wulf Eskildsen^4^, Peter Svensson^1,2,5^

^1^Section of Orofacial Pain and Jaw Function, Institute of Odontology and Oral Health, Aarhus University, Denmark

^2^Scandinavian Center for Orofacial Neurosciences (SCON)

^3^Department of Radiology, Aarhus University Hospital, Denmark

^4^Department of Neuroradiology, Aarhus University Hospital, Denmark

^5^Department of Dental Medicine, Karolinska Institutet, Huddinge, Sweden

**Corresponding author:**

Simple Futarmal Kothari, BDS, PhD student

Section of Orofacial Pain and Jaw Function

Institute of Odontology and Oral Health, Aarhus University

Vennelyst Boulevard 9

DK-8000, Aarhus C, Denmark.

Email: [simple.futarmal@odont.au.dk](mailto:simple.futarmal@odont.au.dk)

Phone number: +45 5039 5697

Fax number: +45 8942 4297

| Table S1. RDC/TMD axis I clinical diagnoses of the TMD pain patients at the most  and less painful TMJ | | | | | | |
| --- | --- | --- | --- | --- | --- | --- |
|  | | Number of patients  (most painful side; n=58) | | | Number of patients  (less painful side; n=58) | |
| RDC/TMD axis I diagnoses | | n | % | n | | % |
| I  II  IIIa  IIIb  I+II  I+IIIa  I+IIIb  II+IIIa  II+IIIb  I+II+III  No diagnosis | 47  23  43  15  21  34  13  16  6  20  0 | | 81.0  39.6  74.1  25.8  36.2  58.6  22.4  27.6  10.3  34.5  0.0 | 47  8  20  3  7  17  2  5  0  4  10 | | 81.0  13.8  34.5  5.2  12.1  29.3  3.4  8.6  0.0  6.9  17.2 |

RDC/TMD = Research Diagnostic Criteria for temporomandibular disorders;

TMJ = temporomandibular joint

| Table S2. LossGain distribution in healthy reference controls and TMJ arthralgia and osteoarthritis patients diagnosed after each and combined examination modalities. | | | | | | |
| --- | --- | --- | --- | --- | --- | --- |
| Loss | | Gain | | | | |
|  | | G0 (No) | G1 (thermal) | G2 (mechanical) | G3 (both) | All |
| **Reference (n = 41)** | |  |  |  |  |  |
| L0 (No) | | 27 (65.6%) | 0 (0.0%) | 5 (12.2%) | 1 (2.4%) | 32 (77.8%) |
| L1 (thermal) | | 2 (4.9%) | 0 (0.0%) | 2 (4.9%) | 0 (0.0%) | 4 (9.7%) |
| L2 (mechanical) | | 1 (2.4%) | 2 (4.9%) | 0 (0.0%) | 1 (2.4%) | 5 (12.2%) |
| L3 (both) | | 0 (0.0%) | 0 (0.0%) | 0 (0.0%) | 0 (0.0%) | 0 (0.0%) |
| All |  | 30 (72.0%) | 2 (4.9%) | 7 (17.0%) | 2 (4.9%) | 41 (100%) |
|  |  |  |  |  |  |  |
| **Clinical diagnosis** |  |  |  |  |  |  |
| *Arthralgia patients (n = 43)* | |  |  |  |  |  |
| L0 (No) |  | 2 (4.6%) | 1 (2.3%) | 16 (37.1%) | 4 (9.3%) | 23 (53.4%) |
| L1 (thermal) |  | 0 (0.0%) | 0 (0.0%) | 4 (9.3%) | 3 (6.9%) | 7 (16.2%) |
| L2 (mechanical) |  | 1 (2.3%) | 0 (0.0%) | 2 (4.6%) | 6 (13.9%) | 9 (20.9%) |
| L3 (both) |  | 0 (0.0%) | 1 (2.3%) | 3 (6.9%) | 0 (0.0%) | 4 (9.3%) |
| All |  | 3 (6.9%) | 2 (4.6%) | 25 (58%) | 13 (30.2%) | 43 (100%) |
|  |  |  |  |  |  |  |
| *OA patients (n =15)* |  |  |  |  |  |  |
| L0 (No) |  | 0 (0.0%) | 1 (6.7%) | 2 (13.3%) | 2 (13.3%) | 5 (33.3%) |
| L1 (thermal) |  | 0 (0.0%) | 0 (0.0%) | 2 (13.3%) | 0 (0.0%) | 2 (13.3%) |
| L2 (mechanical) |  | 0 (0.0%) | 0 (0.0%) | 4 (26.6%) | 3 (20%) | 7 (46.6%) |
| L3 (both) |  | 0 (0.0%) | 0 (0.0%) | 0 (0.0%) | 1 (6.7%) | 1 (6.7%) |
| All |  | 0 (0.0%) | 1 (6.7%) | 8 (53.3%) | 6 (40.0%) | 15 (100%) |
|  |  |  |  |  |  |  |
| **CBCT imaging** |  |  |  |  |  |  |
| *Arthralgia patients (n = 28*) | |  |  |  |  |  |
| L0 (No) |  | 1 (3.6%) | 1 (3.6%) | 9 (32.1%) | 3 (10.7%) | 14 (50%) |
| L1 (thermal) |  | 0 (0.0%) | 0 (0.0%) | 3 (10.7%) | 0 (0.0%) | 3 (10.7%) |
| L2 (mechanical) |  | 1 (3.6%) | 0 (0.0%) | 3 (10.7%) | 4 (14.3%) | 8 (28.6%) |
| L3 (both) |  | 0 (0.0%) | 1 (3.6%) | 2 (7.1 %) | 0 (0.0%) | 3 (10.7%) |
| All |  | 2 (7.1 %) | 2 (7.1 %) | 17 (60.7%) | 7 (25%) | 28 (100%) |
|  |  |  |  |  |  |  |
| *OA patients (n = 30)* |  |  |  |  |  |  |
| L0 (No) |  | 1 (3.3%) | 1 (3.3%) | 9 (30.0%) | 2 (6.7%) | 13 (43.3%) |
| L1 (thermal) |  | 0 (0.0%) | 0 (0.0%) | 4 (13.3%) | 3 (10.0%) | 7 (23.3%) |
| L2 (mechanical) |  | 0 (0.0%) | 0 (0.0%) | 3 (10.0%) | 5 (16.7%) | 8 (26.6%) |
| L3 (both) |  | 0 (0.0%) | 0 (0.0%) | 1 (3.3%) | 1 (3.3%) | 2 (6.7%) |
| All |  | 1 (3.3%) | 1 (3.3%) | 17 (56.6%) | 11 (36.3%) | 30 (100%) |
|  |  |  |  |  |  |  |
| **MRI** |  |  |  |  |  |  |
| *Arthralgia patients (n = 44)* | |  |  |  |  |  |
| L0 (No) |  | 2 (4.5%) | 1 (2.3%) | 15 (34.1%) | 3 (6.9%) | 21 (47.7%) |
| L1 (thermal) |  | 0 (0.0%) | 0 (0.0%) | 4 (9.1%) | 3 (6.9%) | 7 (15.9%) |
| L2 (mechanical) |  | 1 (2.3%) | 0 (0.0%) | 4 (9.1%) | 8 (18.2%) | 13 (29.5%) |
| L3 (both) |  | 0 (0.0%) | 1 (2.3%) | 2 (4.5%) | 0 (0.0%) | 3 (6.9%) |
| All |  | 3 (6.9%) | 2 (4.5%) | 25 (56.8%) | 14 (31.8%) | 44 (100%) |
|  |  |  |  |  |  |  |
| *OA patients (n = 14)* |  |  |  |  |  |  |
| L0 (No) |  | 0 (0.0%) | 1 (7.1%) | 3 (21.4%) | 2 (14.3%) | 6 (42.8%) |
| L1 (thermal) |  | 0 (0.0%) | 0 (0.0%) | 3 (21.4%) | 0 (0.0%) | 3 (21.4%) |
| L2 (mechanical) |  | 0 (0.0%) | 0 (0.0%) | 2 (14.3%) | 1 (7.1%) | 3 (21.4%) |
| L3 (both) |  | 0 (0.0%) | 0 (0.0%) | 1 (7.1%) | 1 (7.1%) | 2 (14.3%) |
| All |  | 0 (0.0%) | 1 (7.1%) | 9 (64.3%) | 4 (28.6%) | 14 (100%) |
|  |  |  |  |  |  |  |
| **HR-US** |  |  |  |  |  |  |
| Arthralgia patients (n = 28) | |  |  |  |  |  |
| L0 (No) |  | 0 (0.0%) | 1 (3.6%) | 6 (21.4%) | 2 (7.1 %) | 9 (32.1%) |
| L1 (thermal) |  | 0 (0.0%) | 0 (0.0%) | 4 (14.3%) | 1 (3.6%) | 5 (17.9%) |
| L2 (mechanical) |  | 0 (0.0%) | 0 (0.0%) | 4 (14.3%) | 6 (21.4%) | 10 (35.7%) |
| L3 (both) |  | 0 (0.0%) | 1 (3.6%) | 2 (7.1 %) | 1 (3.6%) | 4 (14.3%) |
| All |  | 0 (0.0%) | 2 (7.1 %) | 16 (57.1%) | 10 (35.7%) | 28 (100%) |
|  |  |  |  |  |  |  |
| *OA patients (n = 30)* |  |  |  |  |  |  |
| L0 (No) |  | 2 (6.7%) | 1 (3.3%) | 12 (39.6%) | 3 (10.0%) | 18 (60.0%) |
| L1 (thermal) |  | 0 (0.0%) | 0 (0.0%) | 3 (10.0%) | 2 (6.7%) | 5 (10.0%) |
| L2 (mechanical) |  | 1 (3.3%) | 0 (0.0%) | 2 (6.7%) | 3 (10.0%) | 6 (20.0%) |
| L3 (both) |  | 0 (0.0%) | 0 (0.0%) | 1 (3.3%) | 0 (0.0%) | 1 (3.3%) |
| All |  | 3 (10.0%) | 1 (3.3%) | 18 (60.0%) | 8 (26.6%) | 30 (100%) |
|  |  |  |  |  |  |  |
| **Combined** |  |  |  |  |  |  |
| *Arthralgia patients (n = 15)* | |  |  |  |  |  |
| L0 (No) |  | 0 (0.0%) | 1 (6.7%) | 4 (26.6%) | 2 (13.3%) | 7 (46.6%) |
| L1 (thermal) |  | 0 (0.0%) | 0 (0.0%) | 1 (6.7%) | 0 (0.0%) | 1 (6.7%) |
| L2 (mechanical) |  | 0 (0.0%) | 0 (0.0%) | 2 (13.3%) | 3 (20.0%) | 5 (33.3%) |
| L3 (both) |  | 0 (0.0%) | 1 (6.7%) | 1 (6.7%) | 0 (0.0%) | 2 (13.3%) |
| All |  | 0 (0.0%) | 2 (13.3%) | 8 (53.3%) | 5 (33.3%) | 15 (100%) |
|  |  |  |  |  |  |  |
| *OA patients (n = 9)* |  |  |  |  |  |  |
| L0 (No) |  | 0 (0.0%) | 1 (11.1%) | 3 (33.3%) | 2 (22.2%) | 6 (66.6%) |
| L1 (thermal) |  | 0 (0.0%) | 0 (0.0%) | 1 (11.1%) | 0 (0.0%) | 1 (11.1%) |
| L2 (mechanical) |  | 0 (0.0%) | 0 (0.0%) | 1 (11.1%) | 1 (11.1%) | 2 (22.2%) |
| L3 (both) |  | 0 (0.0%) | 0 (0.0%) | 0 (0.0%) | 0 (0.0%) | 0 (0.0%) |
| All |  | 0 (0.0%) | 1 (11.1%) | 5 (55.5%) | 3 (33.3%) | 9 (100%) |

TMJ = temporomandibular joint; OA = osteoarthritis; CBCT = Cone beam computed tomography, MRI = Magnetic resonance imaging; HR-US = high resolution ultrasonography.

| Table S3. Absolute values of each QST parameter at test and control site in healthy controls and TMJ arthralgia and osteoarthritis patients diagnosed after each and combined examination modality. | | | | | | | | | | | | | | | | | |
| --- | --- | --- | --- | --- | --- | --- | --- | --- | --- | --- | --- | --- | --- | --- | --- | --- | --- |
|  |  | | | **CDT** | | **WDT** | | **TSL** | | **CPT** | **HPT** | **MDT** | **MPT** | **MPS** | **WUR** | **VDT** | **PPT** |
| **Reference** | |  | | | |  | |  | |  |  |  |  |  |  |  |  |
| Test site | | 29.9 (1.1) | | | | 34.3 (1.1) | | 36.4 (2.3) | | 13.8 (8.1) | 43.3 (3.5) | 0.39 (0.3) | 251.6 (131.7) | 6.4 (5.8) | 2.6 (1.5) | 7.1 (0.7) | 178.7 (37.5) |
| Control site | | 29.7 (1.1) | | | | 34.1 (0.7) | | 36.1 (1.8) | | 11.1 (7.9) | 43.7 (2.9) | 0.42 (0.4) | 262.0 (124.2) | 6.3 (6.3) | 2.2 (1.1) | 7.2 (0.6) | 177.8 (40.8) |
| **Clinical Diagnosis** | | | | | | | | | | | | | | | | | |
| Arthralgia  Test site  Control site  Osteoarthritis  Test site  Control site | | |  | |  | |  | |  | |  |  |  |  |  |  |  |
|  |  |  | 29.5 (1.7) | | 34.9 (1.5) | | 37.4 (3.7) | | 14.7 (9.5) | | 42.8 (4.0) | 0.67 (0.9) | 204.3 (176.2) | 9.5 (9.7) | 3.2 (3.1) | 7.1 (0.7) | 120.2 (45.7) |
|  |  |  | 29.7 (1.2) | | 34.6 (2.0) | | 36.9 (2.3) | | 13.4 (8.6) | | 42.9 (3.9) | 0.42 (0.4) | 196.3 (167.3) | 12.8 (27.5) 3.7 (4.8) 7.1 (0.7) | | | 133.7 (38.7) |
|  |  |  |  | |  | |  | |  | |  |  |  |  |  |  |  |
|  |  |  | 29.6 (2.4) | | 35.2 (3.7) | | 37.5 (5.8) | | 18.7 (9.9) | | 41.5 (4.7) | 0.87 (1.0) | 164.5 (141.6) | 9.3 (9.9) | 3.1 (2.0) | 6.7 (0.6) | 106.7 (42.0) |
|  |  |  | 29.6 (1.5) | | 34.5 (1.6) | | 36.8 (3.3) | | 17.7 (9.4) | | 42.4 (3.9) | 0.49 (0.5) | 126.3 (96.8) | 10.6 (12.9) 2.3 (1.2) | | 6.9 (0.5) | 124.8 (42.5) |
| **CBCT imaging** | | | | | | | | | | | | | | | | | |
| Arthralgia | | |  | |  | |  | |  | |  |  |  |  |  |  |  |
| Test site | | | 29.7 (1.3) | | 34.9 (1.4) | | 37.8 (3.1) | | 16.1 (9.3) | | 42.7 (3.6) | 0.89 (1.3) | 219.3 (178.4) | 9.6 (9.9) | 3.8 (3.6) | 7.0 (0.7) | 114.4 (39.9) |
| Control site | | | 29.8 (1.1) | | 34.4 (0.9) | | 37.0 (2.4) | | 14.7 (8.3) | | 42.9 (3.5) | 0.45 (0.5) | 181.8 (138.1) | 16.3(33.3) | 4.5 (5.8) | 7.1 (0.6) | 127.5 (36.6) |
| Osteoarthritis | | |  | |  | |  | |  | |  |  |  |  |  |  |  |
| Test site | | | 29.3 (2.4) | | 35.0 (2.8) | | 37.1 (5.2) | | 15.4 (10.2) | | 42.2 (4.7) | 0.57 (0.5) | 170.4 (156.2) | 9.3 (9.7) | 2.6 (1.8) | 6.9 (0.8) | 118.8 (49.5) |
| Control site | | | 29.5 (1.4) | | 34.7 (2.5) | | 36.7 (2.5) | | 14.3 (9.7) | | 42.8 (4.4) | 0.42 (0.4) | 174.9 (170.8) | 8.6 (10.7) | 2.2 (1.2) | 7.2 (0.6) | 135.1 (42.4) |
| **MRI** | | | | | | | | | | | | | | | | | |
| Arthralgia | | |  | |  | |  | |  | |  |  |  |  |  |  |  |
| Test site | | | 29.6 (1.8) | | 34.8 (1.4) | | 37.4 (3.7) | | 15.3 (9.9) | | 42.8 (3.8) | 0.76 (1.2) | 209.3 (166.2) | 10.0(10.5) | 2.9 (2.8) | 7.0 (0.8) | 120.0 (44.0) |
| Control site | | | 29.7 (1.3) | | 34.6 (2.1) | | 36.9 (2.4) | | 13.6 (8.5) | | 43.1 (3.9) | 0.48 (0.5) | 190.2 (141.4) | 14.3 (27.6) 3.3 (4.7) | | 7.1 (0.7) | 135.3 (36.7) |
| Osteoarthritis | | |  | |  | |  | |  | |  |  |  |  |  |  |  |
| Test site | | | 29.2 (2.2) | | 35.6 (3.8) | | 37.6 (5.8) | | 17.3 (9.2) | | 41.4 (5.3) | 0.62 (0.6) | 145.8 (168.8) | 7.5 (6.5) | 3.8 (2.8) | 6.9 (0.6) | 105.6 (46.9) |
| Control site | | | 29.4 (1.3) | | 34.5 (1.0) | | 36.8 (2.8) | | 18.2 (9.7) | | 41.9 (3.9) | 0.30 (0.2) | 140.6 (191.2) | 6.0 (5.6) | 3.4 (2.8) | 7.2 (0.4) | 119.1 (46.9) |
| **HR-US** | | | | | | | | | | | | | | | | | |
| Arthralgia | | |  | |  | |  | |  | |  |  |  |  |  |  |  |
| Test site | | | 28.9 (2.5) | | 35.6 (3.0) | | 39.0 (5.5) | | 15.4 (9.6) | | 43.1 (4.2) | 1.01 (1.2) | 214.6 (174.8) | 10.0 (10.7) 3.1 (3.1) | | 6.9 (0.7) | 128.6 (44.7) |
| Control site | | | 29.4 (1.5) | | 34.9 (2.5) | | 37.7 (2.9) | | 13.3 (7.6) | | 43.0 (3.7) | 0.56 (0.5) | 191.8 (127.1) | 16.8 (33.6) 3.4 (5.1) | | 6.9 (0.6) | 140.7 (37.1) |
| Osteoarthritis | | |  | |  | |  | |  | |  |  |  |  |  |  |  |
| Test site | | | 30.0 (0.9) | | 34.4 (0.9) | | 35.9 (1.6) | | 16.1 (10.0) | | 41.8 (4.2) | 0.46 (0.5) | 174.8 (161.2) | 8.8 (8.8) | 3.2 (2.7) | 7.0 (0.7) | 105.5 (42.6) |
| Control site | | | 29.9 (1.1) | | 34.2 (0.9) | | 36.0 (1.5) | | 15.6 (9.9) | | 42.6 (4.2) | 0.32 (0.2) | 165.5 (177.6) | 8.0 (9.1) | 3.3 (3.4) | 7.3 (0.5) | 122.7 (40.4) |
| **Combined** | | | | | | | | | | | | | | | | | |
| Arthralgia | | |  | |  | |  | |  | |  |  |  |  |  |  |  |
| Test site | | | 29.4 (1.3) | | 35.2 (1.8) | | 39.1 (3.6) | | 16.0 (9.4) | | 43.2 (3.5) | 1.29 (1.7) | 223.8 (205.6) | 11.0 (11.3) 3.3 (3.6) | | 6.9 (0.9) | 123.9 (41.8) |
| Control site | | | 29.3 (1.2) | | 34.8 (0.1) | | 38.1 (2.5) | | 13.0 (7.6) | | 42.7 (3.7) | 0.65 (0.5) | 178.2 (134.2) | 24.1 (44.0) 4.2 (6.8) | | 6.8 (0.6) | 136.1 (39.9) |
| Osteoarthritis | | |  | |  | |  | |  | |  |  |  |  |  |  |  |
| Test site | | | 30.1 (0.7) | | 34.2 (0.9) | | 35.6 (1.5) | | 18.0 (10.3) | | 39.7 (5.2) | 0.60 (0.7) | 134.2 (181.1) | 9.1 (6.9) | 2.8 (1.5) | 6.9 (0.4) | 105.7 (44.7) |
| Control site | | | 29.6 (1.2) | | 34.2 (0.5) | | 36.3 (1.4) | | 18.8 (9.7) | | 41.2 (4.5) | 0.32 (0.3) | 134.8 (235.5) | 7.2 (5.9) | 2.6 (1.3) | 7.3 (0.3) | 122.2 (57.0) |

Data are presented as mean values, with standard deviation shown in parentheses.

TMJ = temporomandibular joint; QST = quantitative sensory testing; CBCT = Cone beam computed tomography, MRI = Magnetic resonance imaging; HR-US = high resolution ultrasonography; CDT = cold detection threshold (^o^C); WDT = warm detection threshold (^o^C); TSL = thermal sensory limen (^o^C); CPT = cold pain threshold (^o^C); HPT = heat pain threshold (^o^C); MDT = mechanical detection threshold (mN); MPT = mechanical pain threshold (mN); MPS = mechanical pain sensitivity (mean pain rating, 0-100); WUR = windup ratio (ratio of pain rating); VDT = vibration detection threshold (/8); PPT = pressure pain threshold (kPa).
