# Supplementary material for: Pain profiling of patients with temporomandibular joint arthralgia and osteoarthritis diagnosed with different imaging techniques
Source: J Headache Pain. 2016 Jun 27;17(1):61. doi: 10.1186/s10194-016-0653-6 (PMC4923011; doi:10.1186/s10194-016-0653-6)
Supplement: Supplementary file 3 — Supplementary Figures (PPTX 2246 kb) [file 10194_2016_653_MOESM3_ESM.pptx]

## Slide 1
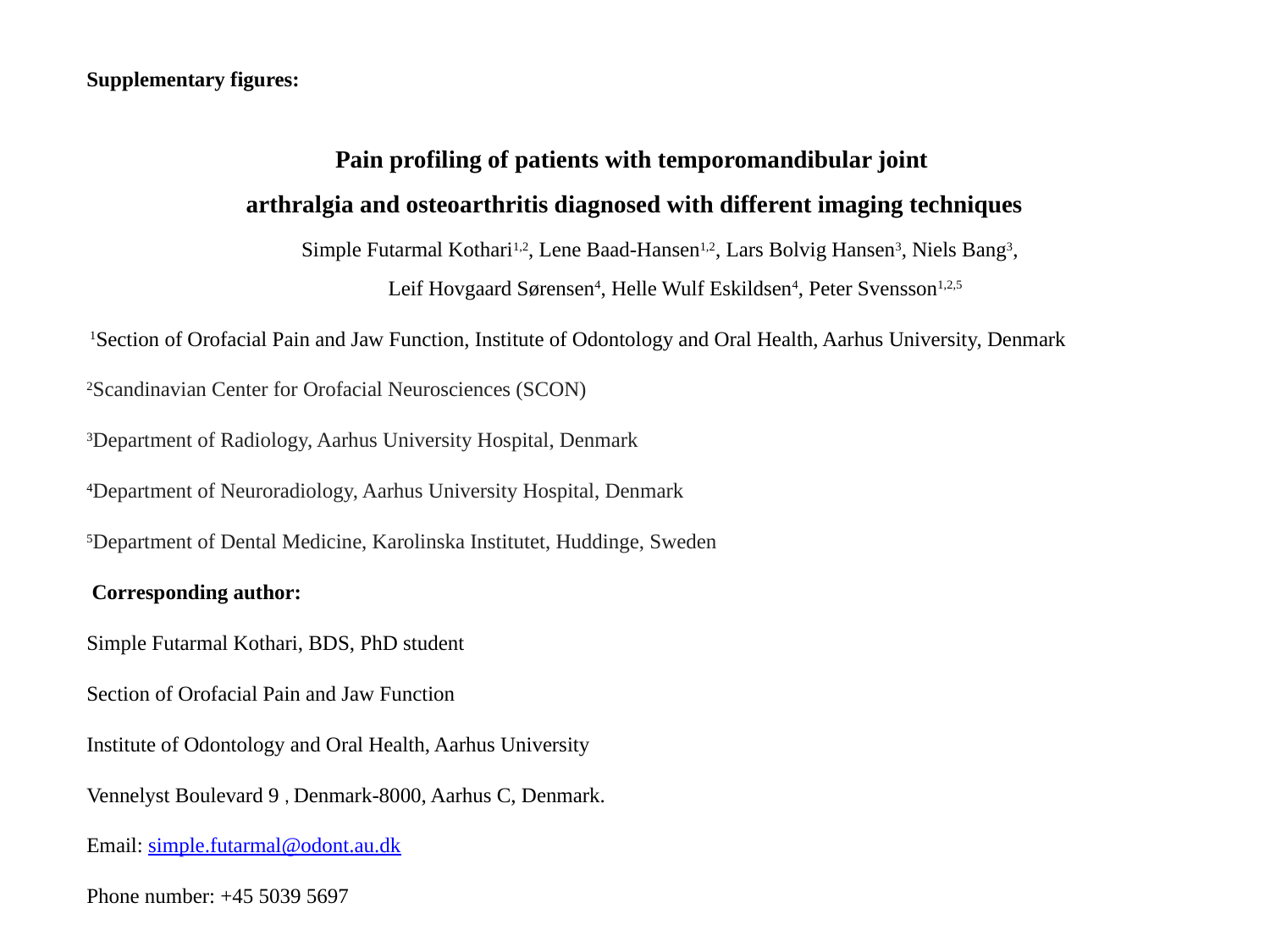

Supplementary figures:
 Pain profiling of patients with temporomandibular joint
 arthralgia and osteoarthritis diagnosed with different imaging techniques
 Simple Futarmal Kothari1,2, Lene Baad-Hansen1,2, Lars Bolvig Hansen3, Niels Bang3,
 Leif Hovgaard Sørensen4, Helle Wulf Eskildsen4, Peter Svensson1,2,5
 1Section of Orofacial Pain and Jaw Function, Institute of Odontology and Oral Health, Aarhus University, Denmark
2Scandinavian Center for Orofacial Neurosciences (SCON)
3Department of Radiology, Aarhus University Hospital, Denmark
4Department of Neuroradiology, Aarhus University Hospital, Denmark
5Department of Dental Medicine, Karolinska Institutet, Huddinge, Sweden
 Corresponding author:
Simple Futarmal Kothari, BDS, PhD student
Section of Orofacial Pain and Jaw Function
Institute of Odontology and Oral Health, Aarhus University
Vennelyst Boulevard 9 , Denmark-8000, Aarhus C, Denmark.
Email: simple.futarmal@odont.au.dk
Phone number: +45 5039 5697

## Slide 2
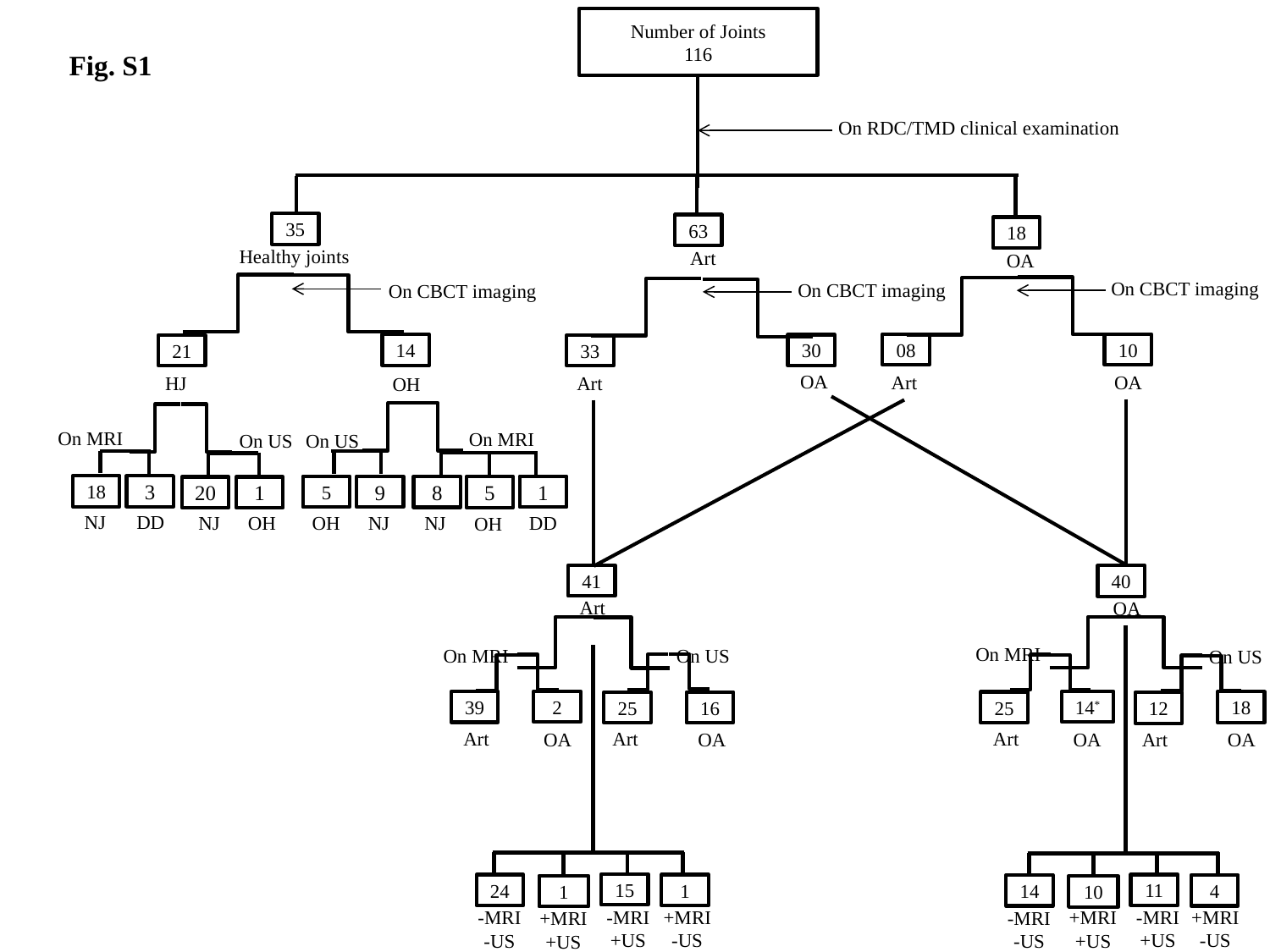

Number of Joints
116
On RDC/TMD clinical examination
35
63
18
Healthy joints
Art
OA
On CBCT imaging
On CBCT imaging
08
10
14
30
21
33
OA
OA
Art
Art
OH
9
8
5
5
On MRI
On US
On US
1
DD
NJ
NJ
NJ
OH
DD
OH
OH
41
On MRI
On US
2
39
16
25
Art
Art
OA
OA
15
1
1
-MRI
+US
+MRI
-US
+MRI
+US
24
-MRI
-US
Art
OA
Fig. S1
On CBCT imaging
HJ
3
20
1
18
On MRI
NJ
40
On MRI
On US
14*
18
25
12
Art
Art
OA
OA
11
4
10
-MRI
+US
+MRI
-US
+MRI
+US
14
-MRI
-US

## Slide 3
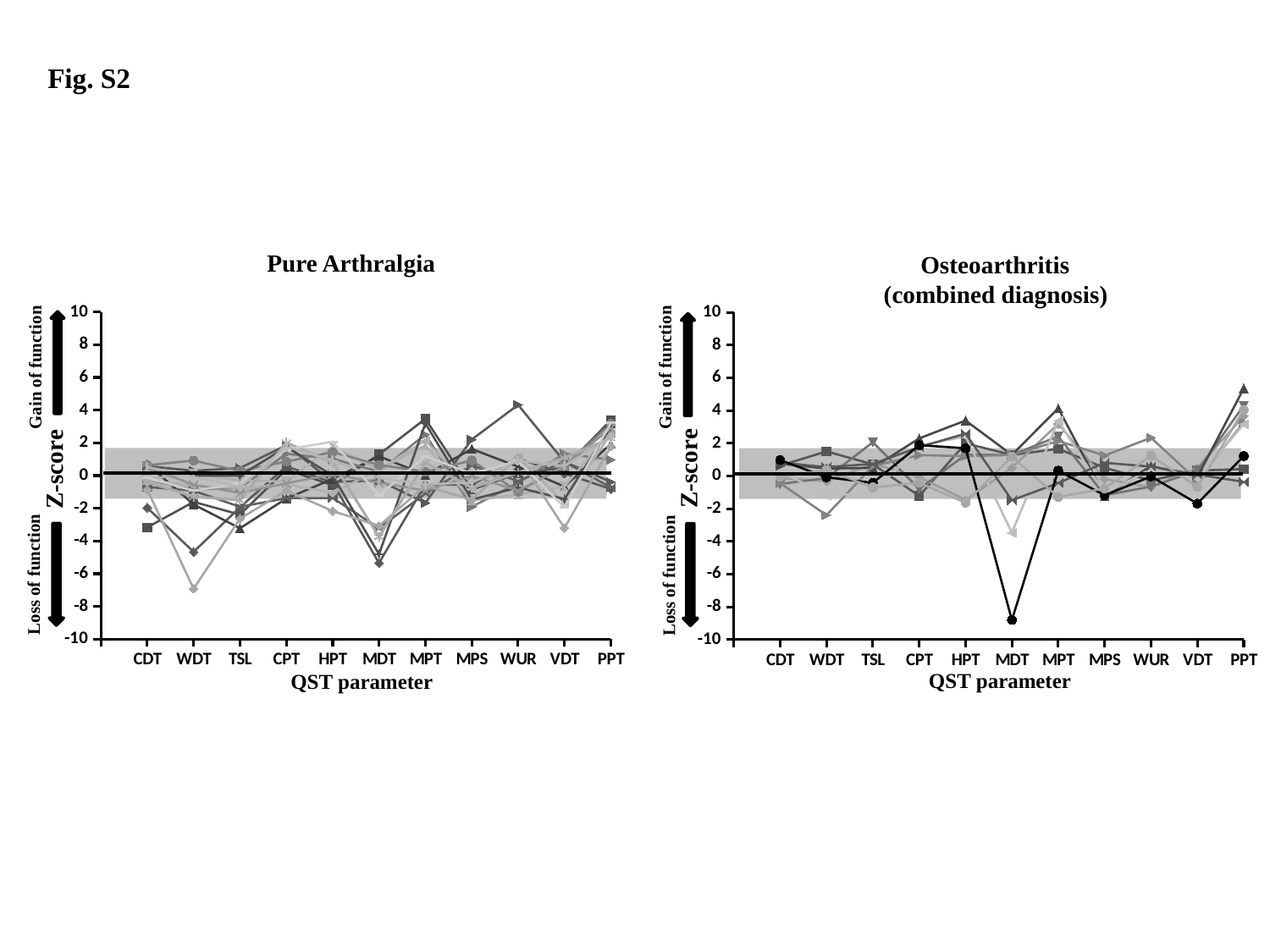

Fig. S2
Pure Arthralgia
 Osteoarthritis
(combined diagnosis)
### Chart
| Category | | | | | | | | | | | | | | | |
|---|---|---|---|---|---|---|---|---|---|---|---|---|---|---|---|
| | None | None | None | None | None | None | None | None | None | None | None | None | None | None | None |
| CDT | -3.168408050560082 | -1.9686608381768629 | 0.5795541755275138 | 0.7060844101978129 | -0.25662133514141455 | -0.2798619897384243 | -0.7023029222584799 | -0.20248523542906086 | 0.6317400619496286 | 0.4523167157517576 | 0.5272483805132365 | -0.8607735879864528 | -0.38863320992790895 | -0.1253705979982558 | 0.5272483805132365 |
| WDT | -1.610729395443809 | -4.664487677227872 | 0.29407476634163465 | -1.7646564048337599 | -0.856699775428642 | -0.6144740243273558 | -0.9290966369051107 | 0.12067698308522232 | 0.9192470370248744 | -0.0027428904895100137 | -0.565861227332661 | -6.919813568381421 | -1.2892256623020872 | -0.9290966369051107 | -0.07662155982474456 |
| TSL | -2.385943593492166 | -1.9393809065995515 | 0.46235895362530766 | -3.221885689011728 | -0.7594396210640667 | -0.772072402580556 | -1.9115267861724579 | -0.30795923488893756 | 0.2820660183454 | -0.005684331756162785 | -1.059692874219326 | -2.630941926742175 | -1.4993591487296374 | -0.47657337828120666 | -0.7038740861336773 |
| CPT | 0.36670901378419796 | 0.5673416304848107 | 1.8571227378458939 | -1.4051114973380976 | 1.3359990581040422 | -0.5270180969730778 | -1.3790553133510053 | -0.3498360458608481 | 0.853959654342829 | 1.7476867651001047 | -0.47099730140082857 | -0.9178608567794665 | 2.00824860497103 | 1.6043777531710957 | 1.760714857093651 |
| HPT | -0.5883831839479998 | -0.42995177257822176 | -0.38613031836956185 | -0.2041027393489684 | 1.0296397406795017 | -1.0603065369643536 | -1.3737984786109316 | 1.7611209378548511 | 1.434145471836375 | -0.0018498737705304118 | 0.07568039136787197 | -2.1726972976457586 | 0.7936780641713236 | 2.064500236222507 | 0.9419968322621793 |
| MDT | 1.285794759869319 | -5.343191264097439 | -0.29675498174027326 | 1.1818539982645202 | 0.24072896564003257 | 0.3689388513156951 | -3.2141215687384235 | 0.3689388513156951 | 0.6542667821199094 | -4.8104128684766625 | -0.43251272083702336 | -3.075603691627225 | -3.741297524683193 | -1.211993359027393 | -0.15067695411175486 |
| MPT | 3.4713322720184467 | -0.5269772167042855 | -1.6694102092119454 | 0.044194978991127946 | 2.5193197616730623 | 2.138791587733256 | -1.0981893510614378 | 1.8529236837708052 | 0.2346148114788227 | 3.185996984265514 | -0.9077845054756248 | -0.6221750433845562 | 0.8058069189363953 | 1.186627321824208 | -0.5269772167042855 |
| MPS | -0.8681273928770875 | -0.568366721517437 | 2.2048171357213135 | 1.6268446796603993 | -1.911446914245751 | -0.8236250166534116 | 0.6169915940449465 | -1.526523495835164 | 0.9246006949203055 | -1.4674909396487428 | -0.020871888601392084 | -1.4851446825661871 | -0.7469056579862203 | 0.11804897439232666 | -0.4339219719413281 |
| WUR | 0.1401972236983711 | 1.024389002606377 | 4.323190027635489 | 0.5432820291215679 | -0.44170571306828355 | 1.1516762867807506 | -0.33307200686473454 | -1.1550261824178263 | -0.9888934196614826 | -0.7330482025121642 | -0.9258877304018822 | 1.1280071067231459 | 0.18686658607384576 | 0.8956993180603718 | 0.9089218230105449 |
| VDT | 0.4798756700596232 | 0.1309760888212057 | 0.8439447983084079 | -0.6730099027281932 | 1.3445398496504863 | -1.6893695524227157 | 0.8439447983084079 | -0.6730099027281932 | 0.5860624991321858 | -1.4314872532464937 | 0.5860624991321858 | -3.206324253459317 | 1.0866575504742642 | -1.9472518515989379 | 0.5860624991321833 |
| PPT | 3.383792361893402 | -0.8237081072831036 | -0.38213094321058744 | 1.9457175931636805 | 0.971661700640099 | 2.5643355776737566 | -0.7289928742969172 | 2.707814053357545 | 3.1655982497759596 | 2.95679179039122 | 2.9059640832632296 | 1.7825425079325663 | 2.289507371186619 | 3.219234173168637 | 2.33424503053261 |
### Chart
| Category | | | | | | | | | |
|---|---|---|---|---|---|---|---|---|---|
| | None | None | None | None | None | None | None | None | None |
| CDT | 0.6317400619496286 | -0.1253705979982558 | -0.4901025531774776 | 0.7505747550444046 | -0.4354090192636887 | 0.30170935726731696 | 0.8245326472594353 | 0.971731473764467 | -0.0485163075768063 |
| WDT | 1.5027196885861582 | -0.3219518821336888 | -0.1258020976425996 | 0.45572882558505534 | -2.398090314421811 | -1.169524527922975 | 0.5430310381183795 | -0.07662155982474456 | 0.3686072295506645 |
| TSL | 0.713855571811469 | 0.7463146427888567 | 2.064631927242858 | 0.4987267298281633 | 0.6037550226085507 | -1.0633915144972765 | 0.7381564543674501 | -0.4184756818119678 | -0.6995431993019792 |
| CPT | -1.2201125910297406 | -0.06712644960089359 | -0.792791173641422 | 2.3000778656264673 | 1.2747670257343744 | 1.8571227378458939 | 1.7659260938910695 | 1.8831789218329864 | -0.4188849334266436 |
| HPT | 1.9970826143630267 | -1.464812268121227 | 1.2993102281174174 | 3.3892565057612725 | 1.2116673197000951 | 2.4117009887988248 | 2.5937285678194186 | 1.693703315995371 | -1.633356322769925 |
| MDT | 1.285794759869319 | 0.5063141216789486 | 1.285794759869319 | 1.285794759869319 | 1.285794759869319 | -3.473395482799729 | -1.48320790648803 | -8.806885098469808 | 1.1818539982645202 |
| MPT | 1.6626386151357697 | 3.185996984265514 | 2.4241868744390143 | 4.137359026340374 | 2.138791587733256 | 3.376199384784399 | -0.4318039520582406 | 0.32979680727884236 | -1.2886051406507126 |
| MPS | 0.4973730385209209 | -0.18009126235170722 | -1.1778481403675385 | -1.213164162590294 | 1.2499623097981467 | -1.0771846522064854 | 0.8052777742145263 | -1.1824734477374148 | -0.7657019824758096 |
| WUR | -0.263594381218891 | -0.6909083713577352 | -0.6495815782326735 | 0.6125101404298047 | 2.336901925997882 | 0.4309396334499922 | 0.574326615994004 | -0.02654441882335997 | 1.2438212033170974 |
| VDT | 0.3281801999559637 | 0.4798756700596232 | 0.3281801999559637 | -0.06622802231355225 | -0.2786016804586773 | -0.17241485138611476 | 0.0854674477901073 | -1.6893695524227157 | -0.6730099027281932 |
| PPT | 0.4264156482744006 | 3.6710952429483283 | 4.3023733457103495 | 5.3519206770711385 | 3.273469393022121 | 3.1655982497759596 | -0.35638021078512194 | 1.2152828047835063 | 4.0399309629329325 |
Gain of function
Gain of function
Z-score
Z-score
Loss of function
Loss of function
QST parameter
QST parameter

## Slide 4
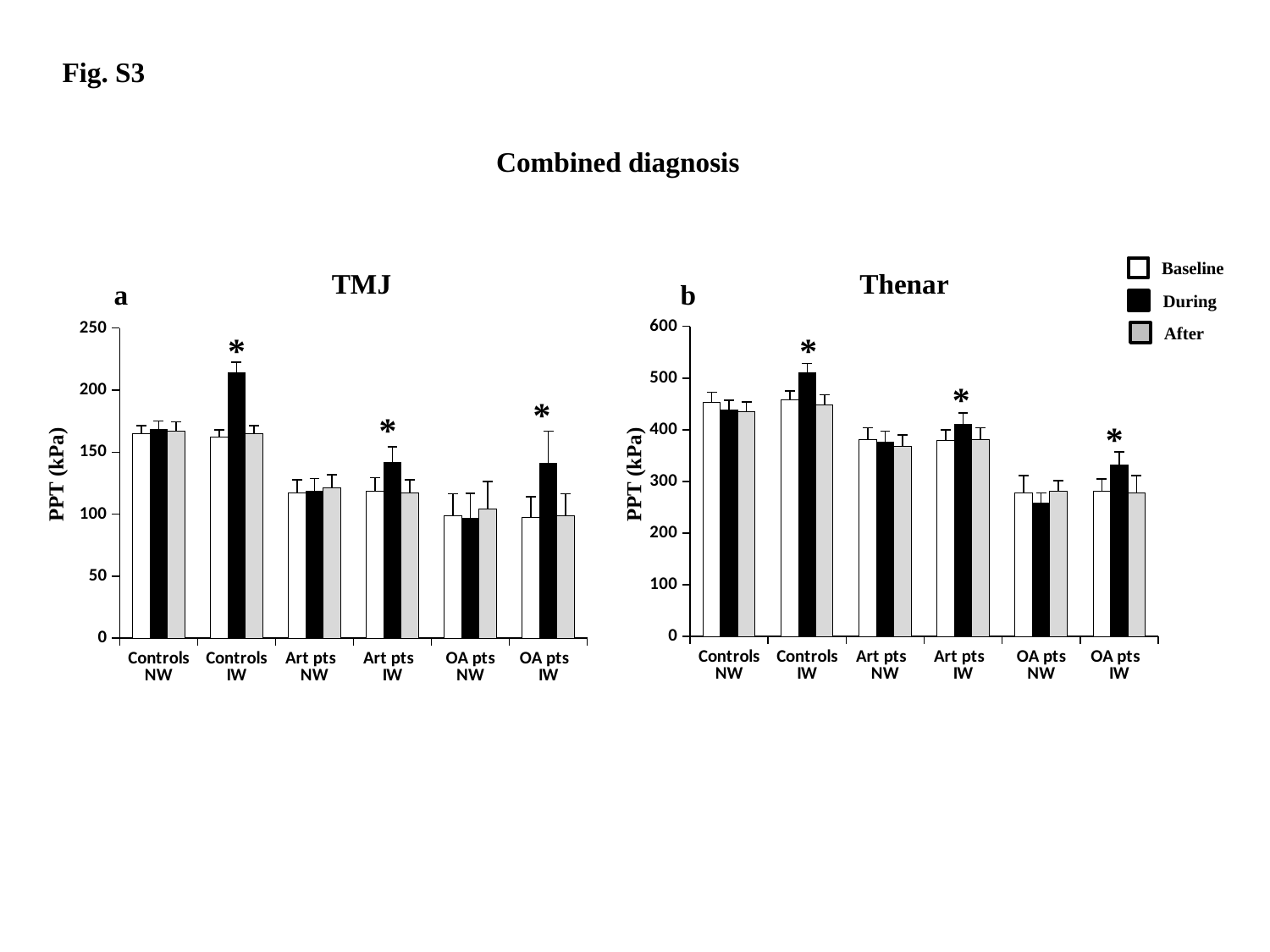

Fig. S3
Combined diagnosis
Baseline
During
After
TMJ
Thenar
a
b
### Chart
| Category | | | |
|---|---|---|---|
| Controls NW | 452.3414634146341 | 438.49593495934954 | 434.8780487804877 |
| Controls IW | 457.4552845528454 | 510.1788617886178 | 448.390243902439 |
| Art pts NW | 381.0 | 375.26190476190476 | 368.19047619047615 |
| Art pts IW | 378.64285714285717 | 411.21428571428567 | 381.0 |
| OA pts NW | 278.38888888888886 | 257.83333333333337 | 280.99999999999994 |
| OA pts IW | 281.1666666666667 | 331.9444444444445 | 278.38888888888886 |
### Chart
| Category | | | |
|---|---|---|---|
| Controls NW | 164.5528455284553 | 168.3821138211382 | 167.1138211382114 |
| Controls IW | 161.9268292682927 | 214.0487804878049 | 164.59349593495935 |
| Art pts NW | 117.33333333333333 | 118.52380952380956 | 120.92857142857143 |
| Art pts IW | 118.16666666666667 | 141.5202380952381 | 117.33333333333333 |
| OA pts NW | 98.77777777777777 | 96.8888888888889 | 104.38889444444446 |
| OA pts IW | 97.0 | 141.11111111111123 | 98.77777777777777 |*
*
*
*
*
*
PPT (kPa)
PPT (kPa)
